# Interactome analysis of transforming growth factor-β-activated kinase 1 in *Helicobacter pylori*-infected cells revealed novel regulators tripartite motif 28 and CDC37

**DOI:** 10.18632/oncotarget.24544

**Published:** 2018-02-21

**Authors:** Olga Sokolova, Thilo Kähne, Kenneth Bryan, Michael Naumann

**Affiliations:** ^1^ Institute of Experimental Internal Medicine, Otto von Guericke University, Magdeburg 39120, Germany; ^2^ EMBL Australia Biomedical Informatics Group, Infection and Immunity Theme, South Australian Health and Medical Research Institute, Adelaide, South Australia 5000, Australia; ^3^ Present address: UCD School of Agriculture and Food Science, University College Dublin, Belfield, Dublin 4, Ireland

**Keywords:** TAB, cytokines, innate immunity, TRIM, STOML2

## Abstract

Transforming growth factor-β (TGFβ)-activated kinase 1 (TAK1) plays a central role in controlling the cellular pro-inflammatory response via the activation of the nuclear factor κB (NF-κB)- and mitogen-activated protein (MAP) kinases-dependent transcriptional programs. Here, we show that depletion of TAK1 and the TAK1-binding proteins TAB1 and TAB2 affects NF-κB, JNK and p38 phosphorylation and suppresses NF-κB activity in AGS cells infected with *Helicobacter pylori* or stimulated with the cytokines TNF and IL-1β. To increase our understanding of TAK1 regulation and function, we performed mass spectrometry (MS)-based TAK1 interactomics. In addition to the identification of known and novel TAK1 interacting proteins, including TRIM28, CDC37 and STOML2, analysis of the MS data revealed various post-translational modifications within the TAK1/TAB complex. By applying siRNAs, TRIM28 and CDC37 were found to regulate phosphorylations of TAK1, IκB kinases IKKα/IKKβ and MAP kinases, NF-κB transactivation activity and IL-8 expression in the infected epithelial cells.

## INTRODUCTION

Transforming growth factor-β (TGFβ)-activated kinase 1 (TAK1) is a mitogen-activated protein (MAP)3 kinase involved in the regulation of cell growth, differentiation and the pro-inflammatory response by activating a range of kinases upstream of nuclear factor κB (NF-κB), ATF-2 and AP-1 transcription factors [1]. In addition to TGFβ, pro-inflammatory cytokines, e.g., IL-1β and TNF, and ligands of toll-like receptors (TLRs), e.g., bacterial endotoxins, induce TAK1. Several genotoxic agents can also trigger TAK1-mediated NF-κB activation, indicating a role of TAK1 in chemotherapy resistance [2]. Dysfunction of TAK1 provides sensitivity to the TNF-induced cell death, and cross-talk between TAK1, RIP kinases and caspase-8 plays here a major role [3]. Thus, TAK1 has emerged as a therapeutic target for inflammatory diseases and cancer [4].

The NF-κB family comprises the RelA, RelB, c-Rel, p100/p52 and p105/p50 proteins in human cells [5]. Binding of NF-κB heterodimers, mainly RelA/p50, to DNA can promote the expression of cell cycle-associated genes and proliferation regulators such as COX-2, c-Myc, cyclins, p21 and p53. NF-κB heterodimers also regulate the expression of the following factors: anti-apoptotic proteins Bcl-2, Bcl-X_L_, and BIRCs; many receptors and cell surface molecules, including epidermal growth factor (GF) receptor (EGFR), glucocorticoid receptor, selectins and N-methyl-D-aspartate receptors; cytokines and growth factors, e.g., IL-8, IL-6, TNF, M-CSF, FAS ligand, and vascular endothelial GF [6]. Aberrant NF-κB signaling has been implicated in a number of diseases, including degenerative disorders, chronic inflammation and cancer [7–9]. The list of NF-κB inducers is impressively long [6]. Nevertheless, TNF, IL-1β and agonists of the pattern recognition receptors are usually used to model NF-κB stimulation. The Gram-negative bacterium *Helicobacter pylori*, which resides in human stomachs, causes gastritis and increases the risk of carcinoma development, is a potent activator of NF-κB in gastric epithelial cells [10]. Common to all inducers of the classical NF-κB pathway is rapid activation of the IκB kinases (IKKs) IKKα, IKKβ and the regulatory protein NEMO, which all exist as a multimolecular complex. Previously, it has been shown that *H. pylori* uses its type 4 secretion system (T4SS) to promote phosphorylation of IKKα and IKKβ [11]. The IKKs phosphorylate the NF-κB inhibitors IκBα, IκBβ, IκBε and thereby mark them for K48-ubiquitinylation and further degradation. In this way, RelA breaks free from an inhibitory cytoplasmic complex, becomes phosphorylated and shuttles to the nucleus in infected cells [12].

The mechanisms of IKKs activation are stimuli-specific and remain a matter of debate [5, 13]. Currently, TAK1 is considered to be an IKKs upstream kinase [14–16]. How TAK1 becomes activated in response to *H. pylori* and its role in gastric cancer development remain unclear. Cytotoxin Associated Gene A (CagA) of *H. pylori* has been suggested to directly bind and regulate TAK1 [17], but this notion has not been confirmed [11].

The aim of our study was to apply a mass spectrometry (MS)-based approach to investigate possible mechanisms of TAK1 activation by identifying TAK1 interacting proteins and their functional role in disease/cancer.

## RESULTS

### TAK1 is constitutively assembled with the adaptor proteins TAB 1-3

**Figure 1:**
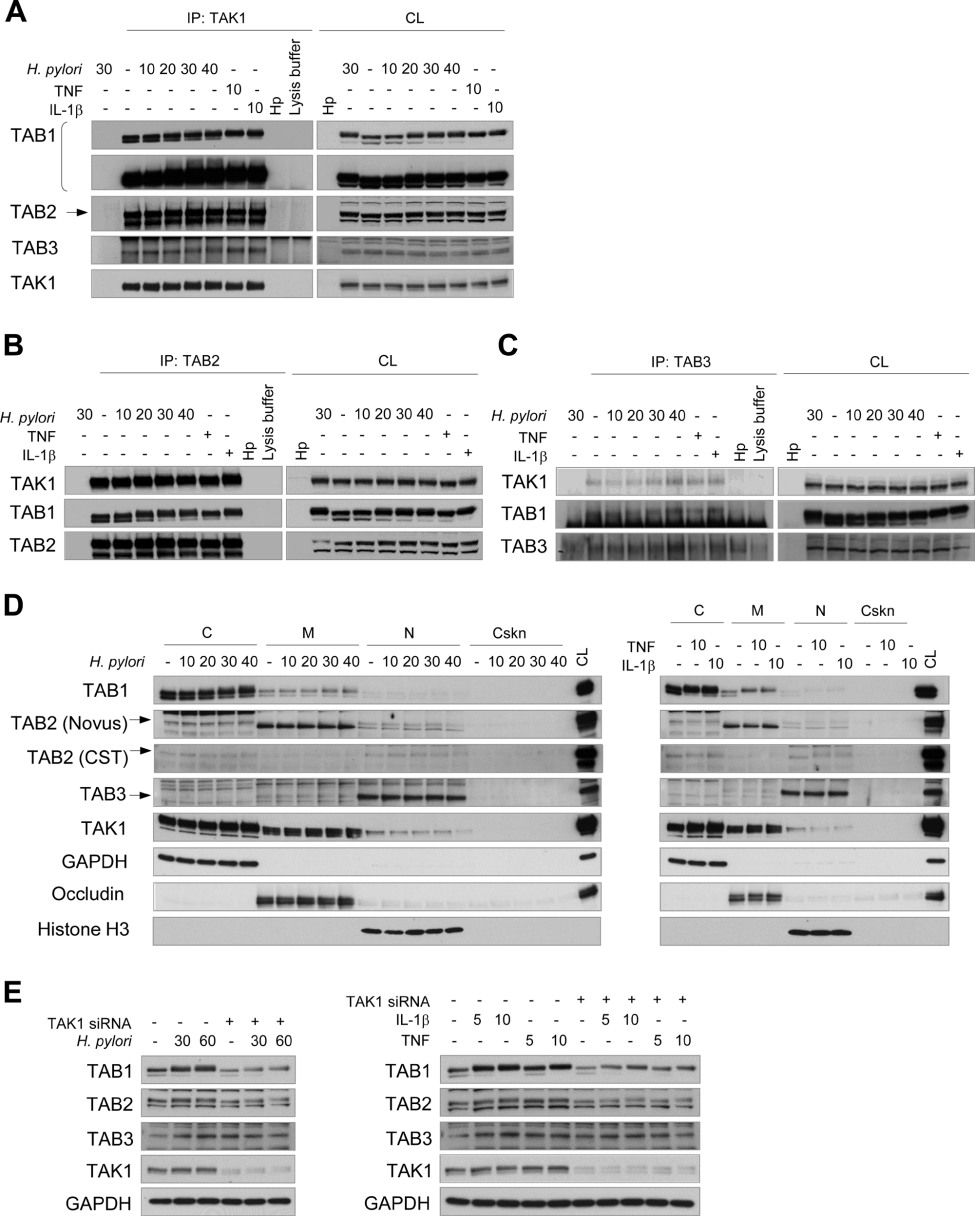
TAK1 constitutively associates with TAB1, TAB2 and TAB3 in AGS cells Immunoblots of TAK1 (**A**), TAB2 (**B**), TAB3 (**C**) immunoprecipitates, subcellular fractions (**D**) and cellular lysates (**E**) are represented. The cells were exposed to *H. pylori*, TNF or IL-1β for the indicated times (in min). *H. pylori* lysate (Hp) was used to demonstrate the absence of antibody cross-reactivity with bacterial proteins. In some lysates of the 30 min-infected cells, the immunoprecipitation antibody was not added to approve non-specific protein binding to the beads. The arrows point specific protein bands. (E) Transfection with TAK1 siRNA was performed 48 h prior to stimulation. C, cytosol, CL, cell lysate; Cskn, cytoskeleton; IP, immunoprecipitation; M, membranes; N, nuclei.

The TAK1-binding proteins TAB1, TAB2 and TAB3 are adaptors of TAK1 that facilitate autophosphorylation of the kinase and assembly of an active TAK1 complex [2]. Co-immunoprecipitation experiments demonstrated that TAB1, TAB2 and TAB3 were constitutively associated with TAK1 in gastric AGS cells (Figure 1A–1C). *H. pylori* infection for up to 40 min, as well as stimulation with TNF and IL-1β for 10 min, did not influence the interactions between TAK1 and TABs. Analysis of the intracellular distribution of TAK1 and TABs revealed that these proteins are present in the cytosol and membranous fractions. The intracellular localization of TAK1 and TABs was unchanged in AGS cells in response to 40 min of infection with *H. pylori* (Figure 1D, left panel) or to 10 min of stimulation with TNF or IL-1β (Figure 1D, right panel). In contrast to Takaesu *et al*. [18], we found no TAB2 translocation from the membranes to the cytosol in response to IL-1β.

Prominent shifts in TAB1 and TAB2 proteins to a lower electrophoretic mobility were detected upon infection with *H. pylori* or stimulation with TNF or IL-1β (Figure 1). The molecular weight shifts of TAB1 and TAB2 may reflect post-translational modifications (PTMs) of these proteins, including multiple phosphorylation events, as has been suggested for TAB1 [19]. Interestingly, TAK1 depletion with siRNA did not prevent the molecular shifts but led to concomitant decreases of TAB1 and TAB2 amounts in AGS cells (Figure 1E). This effect was not due to changes in expression or stability of TAB1 and TAB2 mRNAs, according to qRT-PCR experiments using RNA isolated from AGS cells transfected with scrambled siRNA or with TAK1-specific siRNA (Supplementary Figure 1A). Thus, TAK1 depletion affects the stability of TAB1 and TAB2 proteins, which would provide a degree of balance between TAK1 and TABs in cells.

### TAK1/TABs complex components are crucial for NF-κB activation in *H. pylori*-infected cells

**Figure 2:**
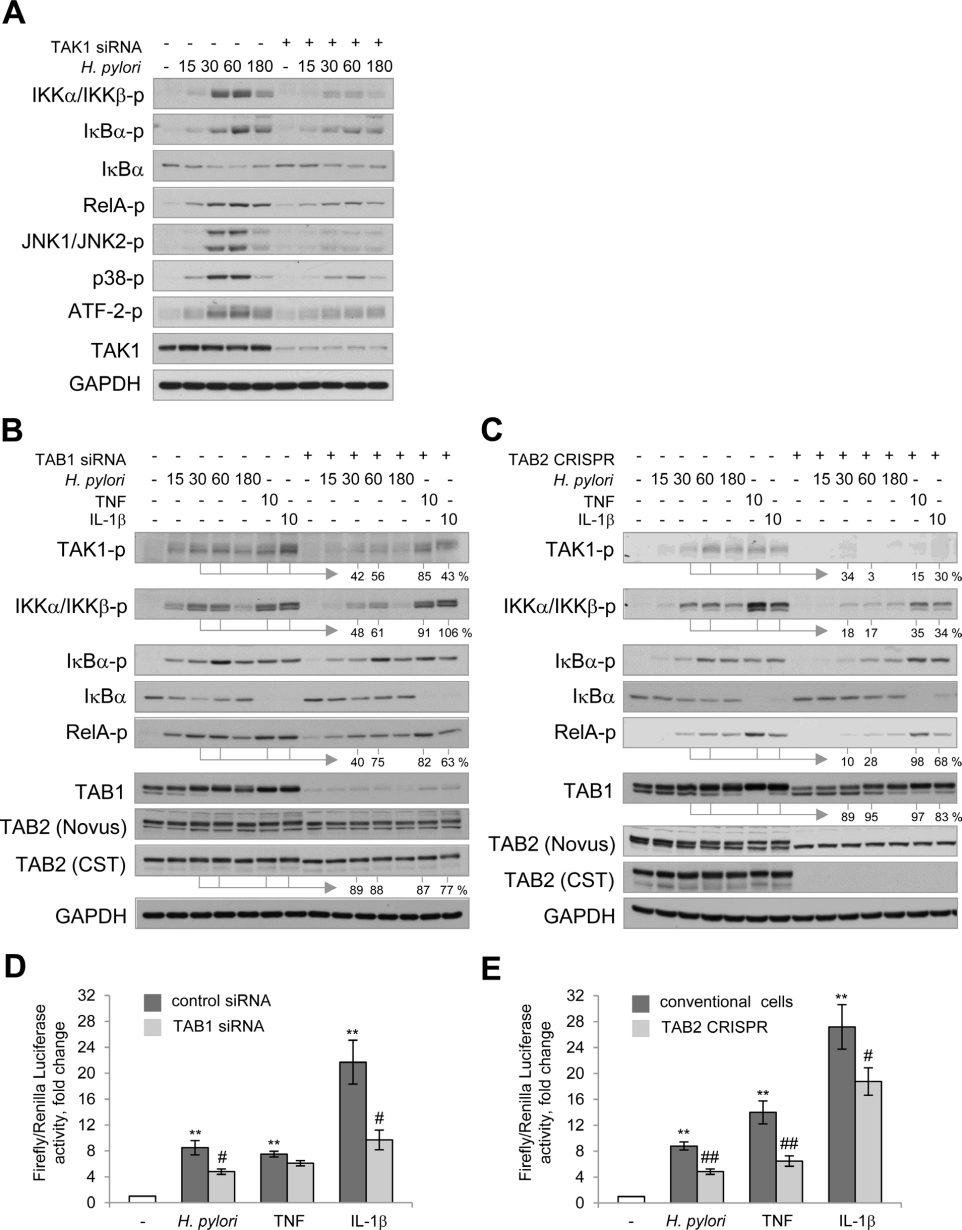
TAK1/TABs complex is required for NF-κB activation in *H. pylori*- and IL-1β-stimulated cells AGS cells were transfected with siRNAs against TAK1 (**A**) and TAB1 (**B**, **D**) or with TAB2 CRISPR/Cas9 and HDR plasmids (**C**, **E**). The cells were exposed to *H. pylori*, TNF or IL-1β for the indicated times (in min). (A–C) Cell lysates were prepared and analyzed via immunoblotting. Densitometry analysis of represented immunoblots was performed. The band intensities (in %) in TAB1 knockdown and TAB2 knockout cells relative to the respective mock values within the infection time course are indicated. (D, E) Cells were transfected with luciferase reporters, treated with *H. pylori* or cytokines and collected with Passive Lysis buffer for the Transactivation assay. ^**^p < 0.01 vs non-stimulated cells, ^#^*p* < 0.05 and ^##^*p* < 0.01 vs stimulated mock cells.

TAK1 coordinates the activity of NF-κB and MAP kinases in a cellular model of *H. pylori* infection. Targeting TAK1 with siRNAs led to a decrease in *H. pylori*-induced phosphorylation of IKKα/IKKβ, their downstream targets IκBα and RelA in AGS cells (Figure 2A). Additionally, TAK1 depletion suppressed phosphorylation of JNK1/JNK2, p38 and one of their downstream targets, the transcription factor ATF-2, within 15–60 min of infection (Figure 2A). A decrease in RelA and MAP kinases activity in TAK1 knockdown epithelial cells has been previously reported to affect the expression of the target gene *CXCL8* (IL-8) in response to *H. pylori* infection [15].

Earlier studies have suggested that TAK1-associated TAB1 and TAB2 mediate downstream pathways in a stimuli- and cell-specific manner, e.g., TAB1 deficiency compromised osmotic stress-induced but not TNF-, IL-1β-, TLR-induced NF-κB and MAP kinase pathways in mouse embryonic fibroblasts [20]. In HeLa cells, TAB1 knockdown affected IL-1α- but not TNF-mediated signaling cascades and release of IL-6 and IL-8 [21]. To evaluate the role of TAB1 and TAB2 in AGS cells, we depleted the proteins using a siRNA pool or CRISPR-Cas9/HDR plasmids, respectively. Deficiency of TAB1 or TAB2 led to impaired phosphorylations of TAK1, IKKα/β, IκBα and RelA in *H. pylori*-infected cells up to about 54% and 18%, and to decrease in TAB2 and TAB1 levels up to about 85% and 91%, respectively (Figure 2B, 2C). The observed decrease in TAB2 and TAB1 amounts was not due to changes at the mRNA level (Supplementary Figure 1B). Further experiments using a luciferase reporter plasmid demonstrated that TAB1 and TAB2 depletion suppressed *H. pylori*-promoted NF-κB transactivation activity (Figure 2D, 2E). Similar effects have been observed in AGS cells stimulated with IL-1β. Interestingly, in the case of TNF, depletion of TAB2 but not TAB1 affected NF-κB signaling (Figure 2B–2E), which suggests that ubiquitin-binding TAB2 may play a predominant role in TNF-induced TAK1 activation in some cell types, including AGS cells.

### TAK1, TAB1 and TAB2 are targets for specific PTMs

**Figure 3:**
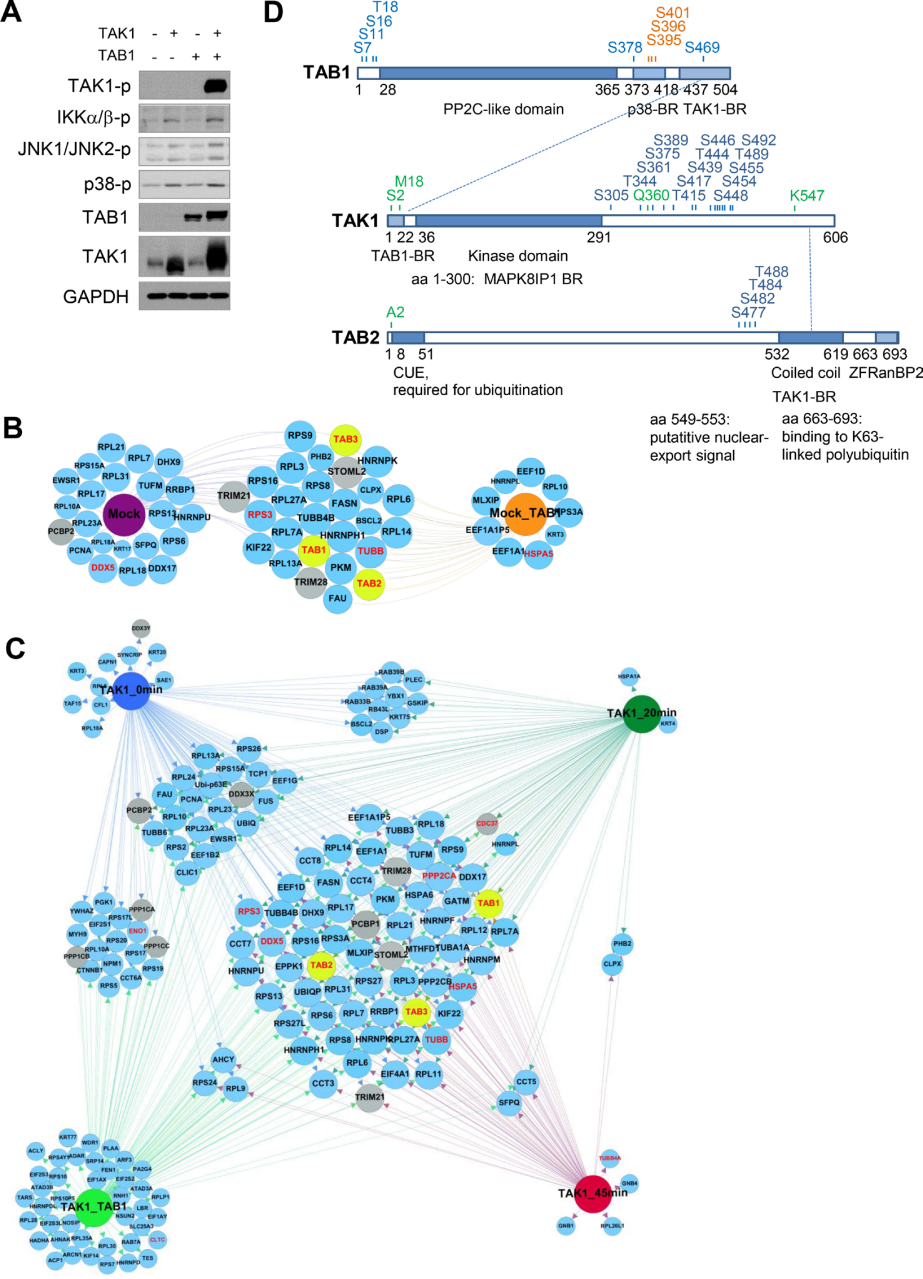
Dynamic TAK1 interactome in AGS cells (**A**) AGS cells were transfected with TAK1 or (and) TAB1 DNA plasmids for 24 h, cellular lysates were prepared from non-starved cells and analyzed by immunoblotting. (**B**) Endogenous TAK1 was immunoprecipitated from mock- or TAB1-transfected AGS cells. (**C**) TAK1 was overexpressed and immunoprecipitated from untreated, *H. pylori*-infected (for 20 and 45 min) and TAB1-co-expessing cells. The samples (B, C) were analyzed by MS. The size of the prey nodes reflects the number of bait interactions, i.e., the smallest nodes have one interaction and the largest interact with all baits. Prey that interact with all baits were positioned at the center of the network; the gray nodes represent novel interactors of interest. TAB1, TAB2 and TAB3 nodes are highlighted in yellow. The already known TAK1 interactors, which have been experimentally validated in human cells and are annotated in the databases InnateDB and IntactDB, are highlighted in red. (**D**) Schematic domain structure of TAK1, TAB1 and TAB2 with phosphorylated (blue), glycosylated (orange) and acetylated (green) sites detected by MS. Interactions between TAK1, TAB1 and TAB2 are shown with dotted lines. BR, binding region; CUE, coupling of ubiquitin conjugation to endoplasmic reticulum; ZF RanBP2, zinc finger domain found in Ran binding protein 2.

To gain further insight into the mechanisms of TAK1 regulation, we overexpressed TAK1 and precipitated the kinase at 0, 20, and 45 min post infection (p.i.) or 48 h post co-transfection with TAB1. We then identified TAK1-associated proteins by MS and analyzed the dynamic interactome using conventional network analyses platforms. According to Kishimoto *et al*., TAK1/TAB1 co-expression activates TAK1 kinase activity [22], which was also observed in AGS cells (Figure 3A). In addition to stabilization and phosphorylation of TAK1, the co-expression led to phosphorylations of IKKα/IKKβ, JNK1/JNK2 and p38, confirming a prominent role of the TAK1/TAB1 complex in regulation of these signaling pathways (Figure 3A). Furthermore, endogenous TAK1 was precipitated from mock- or TAB1-transfected uninfected AGS cells (Figure 3B). MS analysis revealed that TAB1, TAB2 and TAB3 were co-precipitated with endogenous and overexpressed TAK1 in all of the investigated samples (Figure 3B, 3C). TAK1 was phosphorylated on multiple Ser/Thr residues within its C-terminal region, including Ser439, Thr444 and Ser455, and these phosphorylation events were detectable under all conditions (Figure 3D, Supplementary Figure 2A). Infection with *H. pylori* did not change the phosphorylation pattern of TAK1, but co-expression with TAB1 led to phosphorylation of additional sites, e.g., Ser454. The MS analysis also identified phosphorylation sites within TAB1 and TAB2 (Figure 3D). Infection did not change the TAB1 phosphorylation profile, but when overexpressed with TAK1, TAB1 underwent phosphorylation at Ser378 within its p38 kinase-binding region (Figure 3D, Supplementary Figure 3A). Analyzing TAB2 phosphorylation, we found peptides phosphorylated at Ser477, Ser482, Thr484 and Thr488 residues proximal to the TAK1-binding region at 20 min p.i. (Figure 3D, Supplementary Figure 3B). Notably, the phosphorylation sites Ser389, Thr444 of TAK1 and Ser7, Ser378 of TAB1 have been previously identified in the overexpressed proteins [23]. The functional role of the inducible TAB2 phosphorylation remains to be determined.

Glycosylation and acetylation in the context of the TAK1/TABs complex has rarely been investigated. According to our MS data, TAK1 can be acetylated at Met18, which was clearly detectable in samples in which TAK1 stability and activity were supported by TAB1 co-expression (Supplementary Figure 2B). The acetylated region of TAK1 is involved in protein-protein interactions of the kinase, e.g., with TAB1 and MAP kinase 8 interacting protein 1 (MAPK8IP1) (Figure 3D). No acetylated peptides were identified in TAB1. By contrast, TAB2 was constitutively acetylated within an N-terminal (Nt) Ala in both control and infected cells (Supplementary Figure 3B). The Nt Met in nascent proteins is usually cleaved by Met-aminopeptidases if the amino acid at the 2nd position contains an α-amino group. In this way, e.g., Ala becomes the Nt residue and undergoes co-translational acetylation by Nt acetyltransferases [24].

TAB1 was the only glycosylated protein in the TAK1/TABs complex (Figure 3D, Supplementary Figure 3A). In both infected and uninfected cells, O-HexNAcylation (consists of O-GlcNAcylation and O-GalNAcylation) of the TAB1 residues Ser395, Ser396 and Ser401 within the p38-binding part of the protein was detected (Supplementary Figure 3A). O-GlcNac is added to and removed from Ser and Thr residues by O-GlcNAc transferase (OGT) and O-GlcNAcase (OGA), respectively [25]. Overnight incubation of AGS cells with an inhibitor of OGA, PUGNAc (25 μM), increased the amount of glycosylated proteins, including TAB1, in cell lysates (Supplementary Figure 3C). Stimulation with *H. pylori*, IL-1β or TNF promoted TAB1 glycosylation, which was better seen when de-glycosylation was inhibited (Supplementary Figure 3C). Interestingly, TNF, which does not strongly require TAB1 for NF-κB activation (Figure 2D), demonstrated the most prominent effect.

### Interactome analysis revealed novel constitutive and dynamic TAK1 molecular partners

We identified at least 36 (e.g., in the Mock_TAB1 group) TAK1 interactors, which matched to distinct functional groups, including mainly cytoskeleton-related proteins, metabolism regulators, and proteins involved in the translational machinery. The overexpressed TAK1 co-precipitated with ubiquitin (UBIQ) and SUMO-activating enzyme subunit 1 (SAE1), and SAE1 was identified only in non-stimulated cells (Figure 3C). Ubiquitinylation of TAK1 within the TAK1/TABs complex is usually mediated by the E3 ubiquitin ligases TRAF6 (in IL-1β- and *H. pylori*-treated cells) and cIAP1/BIRC2 (in TNF-stimulated cells) [15, 26, 27]. Modifications with SUMO1 at Lys349 have been recently described for TAB2 [28].

PPP2A and PPP2B, which are catalytic subunits of serine/threonine-protein phosphatase 2A (PP2A), were found in TAK1 interactome regardless of stimuli (Figure 3C). Catalytic subunits PPP1CA, PPP1CB and PPP1CC of serine/threonine-protein phosphatase 1 (PP1) were found in non-stimulated or TAB1-co-expressing cells but not in *H. pylori*-infected cells, suggesting a causative role of this type of phosphatase in *H. pylori*-induced TAK1 activation (Figure 3C). Consistently, in the same samples, we detected EIF2S1, NPM1 and YWHAZ (14-3-3-zeta), which have been described as regulatory subunits of PPP1CA, PPP1CB and PPP1CC, respectively [29].

Within the MS data, the chaperoning T complex (CCT), including CCT3, CCT4, CCT6-8 and TCP1, is well represented. This profile is characteristic for some types of cancer cells, whose rapid growth requires up-regulation of chaperones (CDC37, heat shock proteins (HSPs) and CCTs) to prevent the accumulation of misfolded proteins that would trigger apoptosis [30, 31]. Interestingly, CDC37 was enriched in TAK1 immunoprecipitates from AGS cells at 20 min p.i. and from TAK1/TAB1 overexpressing cells (Figure 3C). CDC37 binds to the Nt part of Hsp90 and thereby supports folding and stabilization of a number of Hsp90 interacting proteins, including EGFR, the tyrosine kinases Src and Lck, the serine/threonine kinases Cdk4, Raf-1 and TAK1 [32, 33].

Stomatin-like protein 2 (STOML2) and substrate-recognition subunits of the RING-type E3 ligases Tripartite motif (TRIM)21 and TRIM28 were constitutively associated with TAK1 in all samples analyzed (Figure 3B, 3C). STOML2 can localize in the plasma membrane and is present in abundance in the mitochondrial inner membrane, where it links prohibitins to phospholipid cardiolipin, thereby supporting mitochondrial biogenesis [34]. TRIM21 can act directly as a cytoplasmic Fc receptor, and TRIM28 regulates transcription through binding to DNA, transcription factors, e.g., Oct3/4, NGFI-B, C/EPBb, and KRABs, or recruiting chromatin-modifying enzymes, such as histone methyltransferases [35].

Some nucleic acid-binding proteins, including poly(rC)-binding protein (PCBP)1, its close homolog PCBP2, and Asp-Glu-Ala-Asp (DEAD)-box helicase DDX3, were identified by MS as novel TAK1 interactors (Figure 3B). These proteins are known to function in the cytoplasm, nucleus and in the ribonucleoprotein complex where they regulate gene transcription, RNA splicing and export, ribosome assembly and protein translation. The ability of these proteins to bind RNA suggests their additional role in immunity [36].

### Immunoblotting confirmed TAK1 interaction with TRIM21, TRIM28, STOML2, PCBP2, CDC37, DDX3X and PPP1C

**Figure 4:**
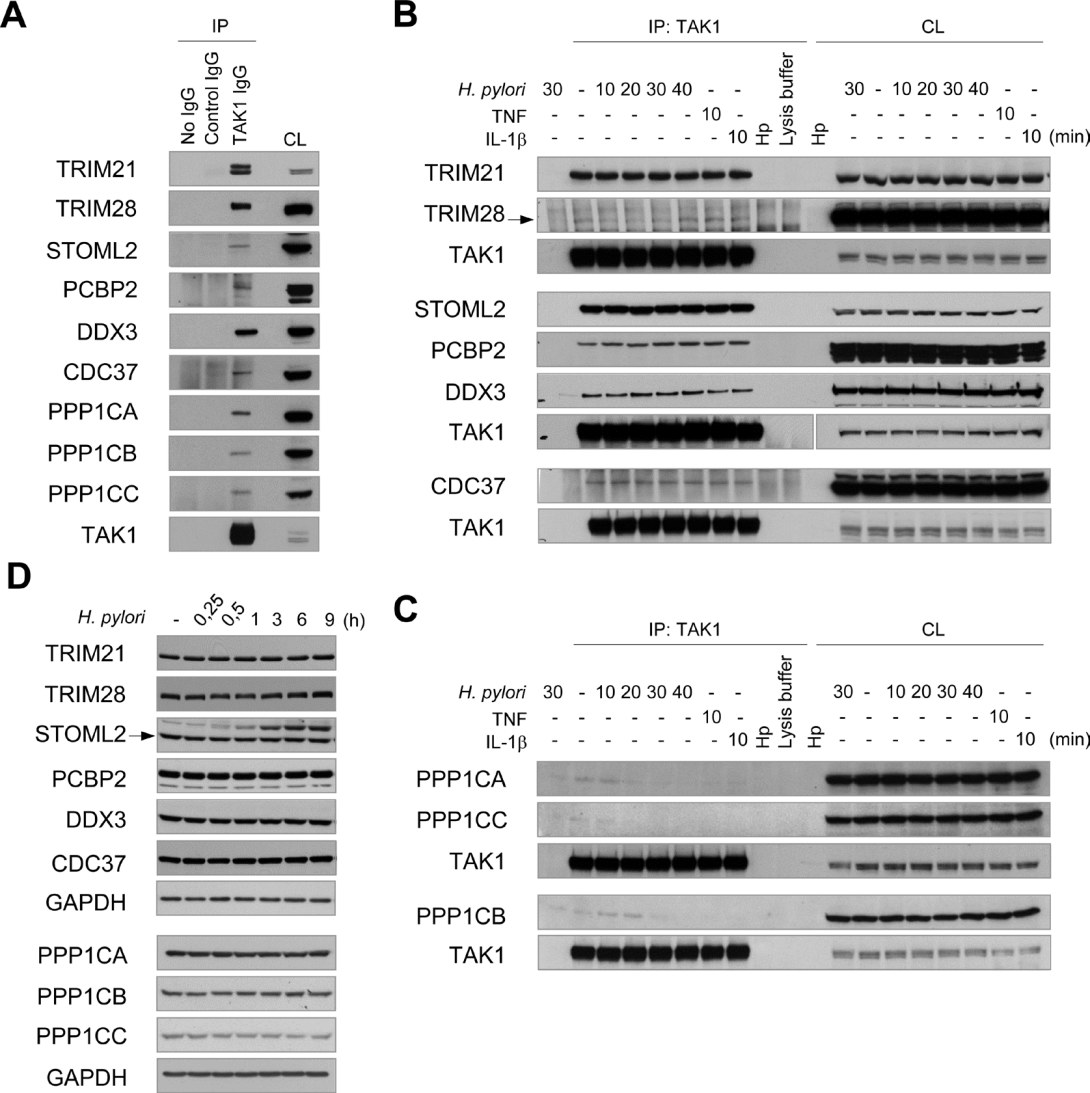
Validation of MS data: TRIM21, TRIM28, STOML2, PCBP2, CDC37, DDX3X and PPP1C interact with TAK1 (**A**) TAK1 was overexpressed and cell lysate (CL) was prepared. Equal aliquots of the lysate were incubated with TAK1 specific antibody or with an isotype control antibody. In one lysate portion, no antibody was added to approve non-specific protein binding to the beads. (**B**, **C**, **D**) AGS cells were treated with *H. pylori*, TNF or IL-1β for the times indicated. TAK1 immunoprecipitates (B, C) and cell lysates (D) were prepared and analyzed by immunoblotting. The arrow points a specific protein band. Samples from different experiments are clearly arranged and indicated by bigger interspaces between the blot panels. (B) The samples detected by TAK1 antibody in the 2nd block originate from one experiment and one gel, but due to different exposure times the panels are separated. CL, cell lysate; Hp, *H. pylori* lysate.

To validate the MS data, TAK1 was overexpressed in AGS cells and immunoprecipitated. Immunoblotting demonstrated constitutive interaction of TRIM21, TRIM28, STOML2, CDC37, PCBP2, DDX3 and PPP1CA with TAK1 (Figure 4A). Furthermore, the interactors were found to be co-precipitated with endogenous TAK1 (Figure 4B). The association of TAK1 with the proteins was not affected within 40 min of infection with *H. pylori* or 10 min of stimulation with TNF or IL-1β (Figure 4B). In agreement with the MS data, infection with *H. pylori* or 10 min of treatment with cytokines led to the dissociation of PPP1CA, PPP1CB and PPP1CC from TAK1 (Figure 4C). The total amounts of TRIMs, STOML2, CDC37, PCBPs, DDX3 and PPP1C isoforms were unchanged in AGS cells within 9 h of infection (Figure 4D).

### TAK1-interacting proteins TRIM28 and CDC37 contribute to TAK1, NF-κB and MAP kinases activation in *H. pylori*-infected cells

**Figure 5:**
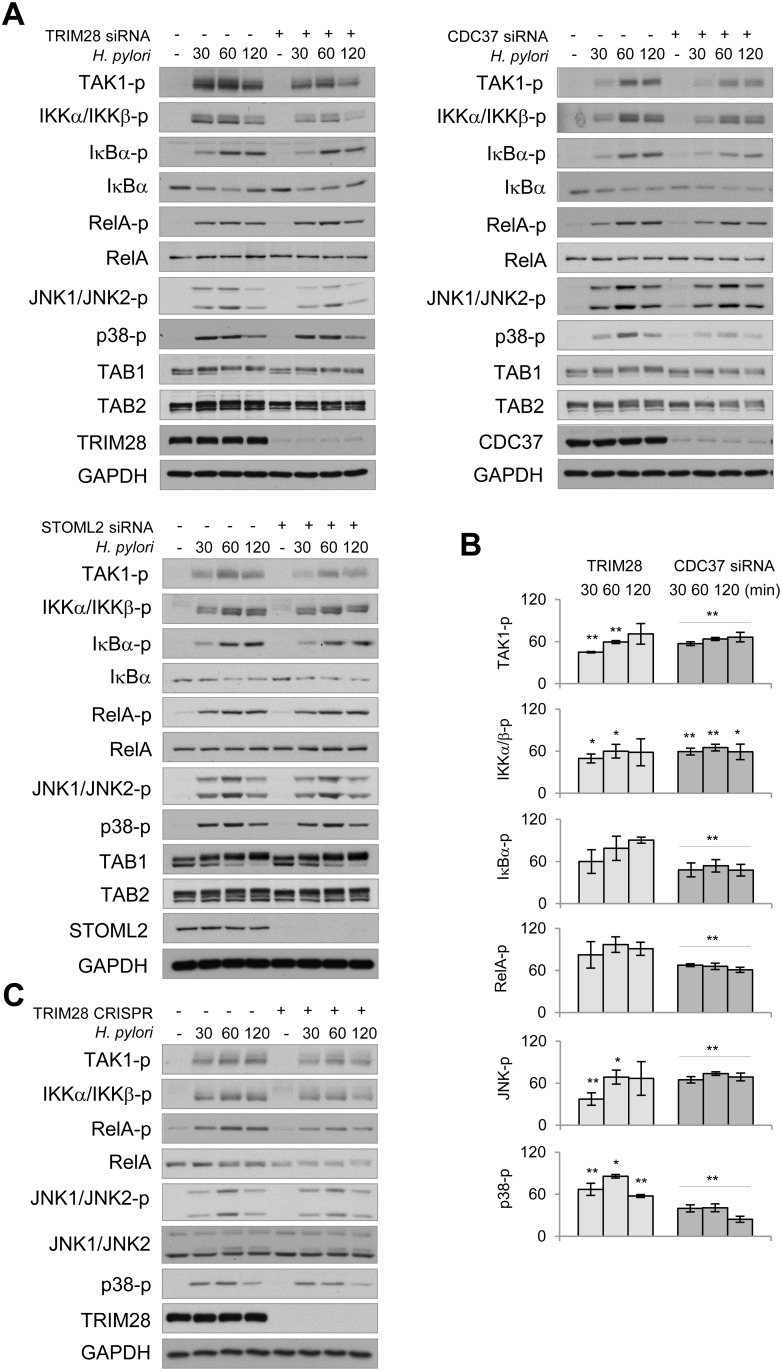
TRIM28 and CDC37 are involved in regulation of NF-κB and MAP kinases in *H. pylori*-infected cells (**A**) Mock- or siRNA-transfected AGS cells were infected with *H. pylori* for the indicated times (in min), and cell lysates were prepared and analyzed by immunoblotting. (**B**) Densitometry analysis of immunoblots from 3 independent experiments was performed. Graphs represent band intensities (in %) in TRIM28 and CDC37 siRNA-transfected cells relative to the respective mock values within the infection time course. (**C**) TRIM28 expressing or TRIM28 knockout AGS cells were infected with *H. pylori* for the indicated times (in min), cell lysates were prepared and analyzed by immunoblotting. ^*^p < 0.05 and ^**^p < 0.01 vs stimulated mock cells.

Some of the novel interactors identified via MS were further investigated for their role in TAK1 regulation. Transient transfection with specific siRNA pools revealed a decrease in *H. pylori*-induced TAK1 autophosphorylation in TRIM28, CDC37, STOML2 (Figure 5A, 5B), TRIM21, DDX3 (Supplementary Figure 4A) and PPP1C knockdown cells (Supplementary Figure 4B). Depletion of TRIM28 and CDC37 led to mitigated phosphorylation of IKKα/IKKβ within 30-120 min of infection (Figure 5A, 5B). In comparison to TRIM28, CDC37 depletion with siRNA had a more prominent effect on IκBα and RelA phosphorylation in infected cells (Figure 5B). However, knockout of TRIM28 by using CRISPR/Cas9 stable transfection led to a dramatic decrease in RelA posphorylation and slight decrease in RelA protein level in AGS cells (Figure 5C). TRIM28 and CDC37 depletion negatively regulated JNK and p38 phosphorylation (Figure 5B). In TRIM 28 knockout cells, *H. pylori*-induced phosphorylation of TAK1, IKKα/IKKβ and MAPKs was comparable with this in TRIM28 siRNA-transfected cells (Figure 5C). In all cases, TAB1 and TAB2 protein shifts and abundances were unaffected. Interestingly, despite the effect on TAK1 autophosphorylation, STOML2 and DDX3 knockdowns failed to affect the *H. pylori*-induced phosphorylation of IKKα/IKKβ, IκBα, RelA and JNK1/JNK2 (Figure 5A, Supplementary Figure 4A). Meanwhile, PPP1C knockdown led to decreased phosphorylation of these proteins in the *H. pylori*-infected cells (Supplementary Figure 4B).

**Figure 6:**
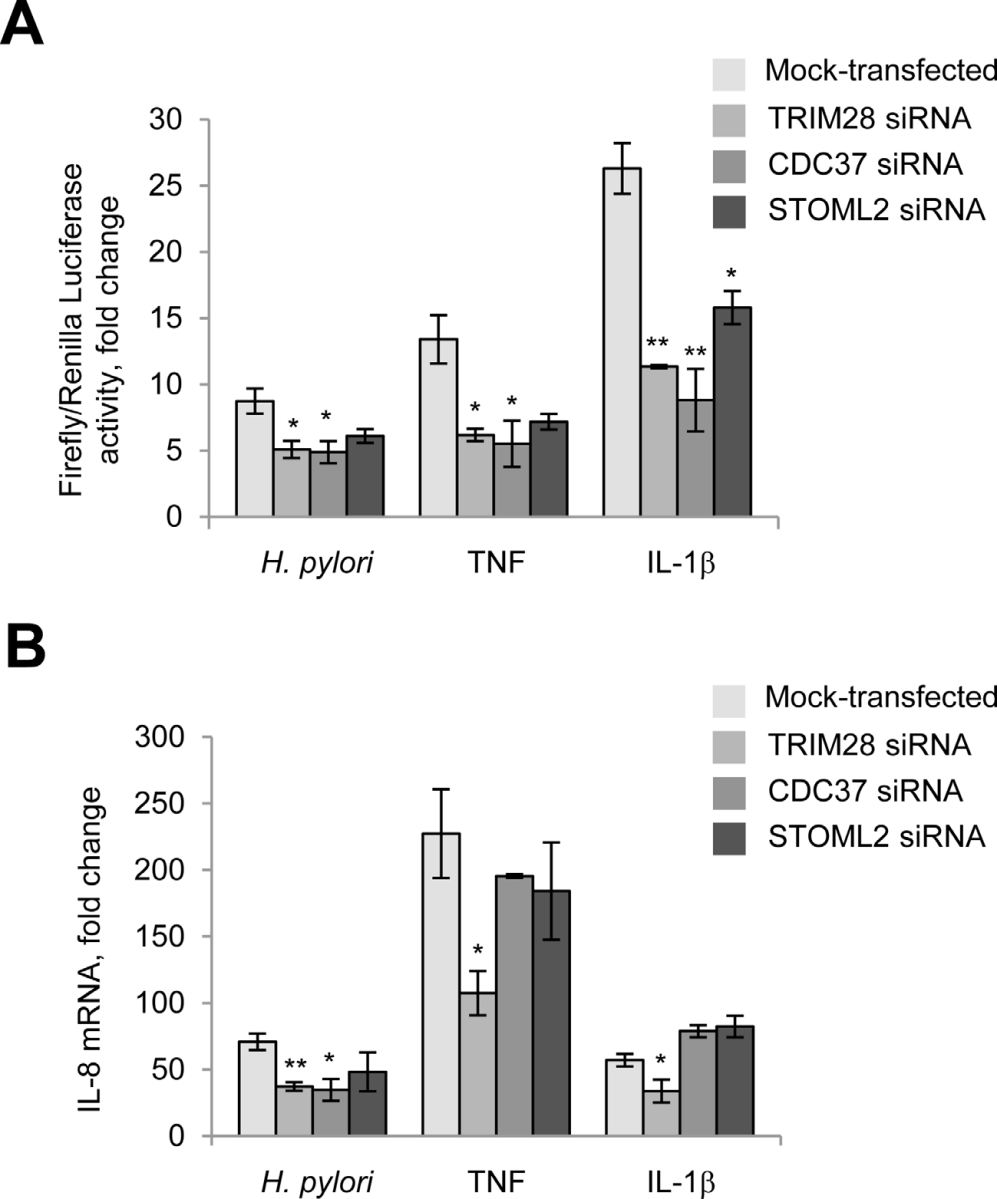
TRIM28 and CDC37 regulate RelA in *H. pylori*-, TNF and IL-1β-stimulated cells. TRIM28 contributes to IL-8 production in response to *H. pylori* (**A**) Cells were transfected with luciferase reporters and siRNAs, treated with *H. pylori* or cytokines and collected with Passive Lysis buffer for the Transactivation assay. (**B**) Cells were transfected with 40 nM of each siRNA for 72 h and then treated with *H. pylori* or cytokines. Total RNA was isolated, and qRT-PCRs were performed. ^*^p < 0.05 and ^**^p < 0.01 vs mock-transfected cells.

Transient reporter assays confirmed that depletion of TRIM28 and CDC37 affected NF-κB activity, in agreement with the immunoblotting data (Figure 6A). Knockdown of STOML2 did not significantly influence NF-κB in *H. pylori*-infected cells (Figure 6A). Furthermore, transient depletion of TRIM28 led to a decrease in IL-8 mRNA in AGS cells at 3.5 h post all stimulations (Figure 6B). Interestingly, in cells transfected with CDC37 siRNA, only *H. pylori*-induced but not TNF- and IL-1β-provoked IL-8 expression was affected. This result might be explained by a regulatory influence of other transcription factors on the IL-8 gene promoter in addition to NF-κB. In TRIM21-, STOML2-, PCBP1-, PCBP2- and DDX3-knockdown AGS cells, IL-8 expression in response to *H. pylori*, TNF and IL-1β did not change significantly (data not shown).

## DISCUSSION

Gastric cancer remains the 3rd leading cause of cancer-related death in the world [37], and *H. pylori* contributes to its pathogenesis by causing chronic gastritis and further gastric metaplasia. In *H. pylori*-infected stomach epithelial cells, the pro-inflammatory response is mediated by a set of signal transduction pathways, including TAK1-IKKs and TAK1-MAP kinases [11, 15, 17]. The exact mechanism of TAK1 activation remains unclear, as well as the role of this kinase in gastric cancer development. We performed MS analysis of TAK1 co-precipitated proteins to decipher their potential role in regulation of TAK1 activity. A constitutive association of TAK1 with the adaptor molecules TAB1, TAB2 and TAB3 in AGS cells was observed. In overexpression experiments by Shibuya *et al*., binding of TAB1 through the last 68 amino acids of the C-terminus to the first 22 Nt amino acids of TAK1 induced a conformational change in TAK1, leading to phosphorylation of both proteins and to an increase in TAK1 kinase activity [38]. TAB2 and TAB3 are known to bind to the same region on the C-terminus of TAK1 [39] and to mediate TAK1 activation by recruiting ubiquitinylated proteins, e.g., ubiquitinylated TRAF6 (IL-1β-induced) or RIP1 (TNF-induced) to the TAK1/TABs complex [40–42]. Our knockdown experiments demonstrated that both TAB1 and TAB2 are important for NF-κB activation in *H. pylori*-infected and IL-1β-stimulated AGS cells. TAB2 but not TAB1 is strongly implicated in TNF-induced NF-κB activation. Interestingly, despite constitutive association with TABs, endogenous TAK1 remains inactive in non-stimulated cells, suggesting that other TAK1-associated molecules, including phosphatases, may modulate the kinase activity. Indeed, TAK1 proteomics revealed that TAK1 is associated with PP2A and PP1, and infection leads to removal of PP1 catalytic subunits from the TAK1 interactome in AGS cells. Dissociation of PP1 from TAK1 in the infected cells was confirmed by immunoblotting analysis. Isoforms of the catalytic subunit of PP2A have been previously described to dephosphorylate TAK1 to control TAK1-mediated signal transduction [43, 44]. In addition, Kim *et al*. demonstrated that the catalytic subunit of PP2A co-precipitated with TAK1 from mice glomerular mesangial cells. There, the PP2A-TAK1 interaction was stimulated by TGFβ, and inhibition of catalytic subunit of PP2A enhanced TAK1 phosphorylation at Thr187 [45]. Interestingly, *H. pylori* strain 99 can induce PP1 and PP2A expression in AGS cells, and their inhibition with okadaic acid augments *H. pylori*-induced activation of MAP kinases and AP-1 [46]. Meanwhile, the gamma catalytic subunit of PP1 has been shown to be necessary for MyD88-dependent TRAF6 activation (via association) and consequent TAK1 autophosphorylation in mouse macrophages and in human monocyte lines [47]. Therefore, phosphatases constitutively interact with and support the function of the TAK1/TABs complex.

*H. pylori* induces protein modifications of TAB1 and TAB2 (detected as a shift to lower electrophoretic mobility by immunoblotting and as inducible TAB2 phosphorylation within amino acids Ser477-Thr488 by MS). According to the literature, phosphorylation of endogenous TABs can be p38α MAP kinase-dependent and provide a negative feedback mechanism to control TAK1 activity [48]. In our experiments, depletion of TAK1 with siRNA prevented *H. pylori*-induced p38 phosphorylation (Figure 2A) but not the TAB1 gel mobility shift (Figure 1E). The mechanism and functional role of the inducible TAB2 phosphorylation within the Ser477-Thr488 region are matters for future investigations.

During the past few years, biochemical studies have provided much evidence that glycosylation and acetylation are of fundamental importance in regulation of protein folding, distribution, stability and activity. Aberrant O-glycosylation, in part via OGT overexpression, is implicated in the pathology of gastric cancer [49–51]. We found that TAB1 is the only O-glycosylated protein in the TAK1/TABs complex. O-GlcNAcylation within amino acids 387–402 has been described previously by Pathak *et al*. [52]. The authors suggested that glycosylation on Ser395 was required for the full activation of TAK1 and NF-κB and for the production of TNF and IL-6 in mouse fibroblasts stimulated by IL-1β and osmotic stress. Similar to O-phosphorylation, O-glycosylation is usually present on small side chain amino acids and proline within intrinsically disordered protein regions and might compete with phosphorylation [53, 54]. According to our data, TAB1 glycosylation can be prominently induced by TNF and may represent an interesting stimuli-specific regulation mechanism of TAK1/TABs activity.

The role of Nt acetylation in protein fate is a debated matter. This acetylation can neutralize the positive charge associated with the α-amino group and thereby protect the N-terminus [55]. By contrast, it has been suggested that a protein containing Nt acetylation is short-lived unless it can shield its N-terminus through intramolecular folding or interactions with other proteins [56]. Thus, constitutive associations of Nt-acetylated TAB2 with other TABs and TAK1 could stabilize the protein, and stoichiometry within the TAK1/TABs complex can influence the kinase activity. Interestingly, knockdown of TAK1 in AGS cells led to decrease of TAB1 and TAB2 on protein level (Figure 1, Supplementary Figure 1). Similarly, we have previously observed that depletion of IKKα and IKKβ affected protein stability of NEMO within the IKK complex [11]. It seems that regulatory/adaptor proteins are stabilized via forming the complex, which contains a specific kinase.

Analysis of the dynamic TAK1 interactome revealed novel TAK1 interacting proteins, namely TRIMs 21 and 28, STOML2, CDC37, PCBPs and DDX3, which are of particular importance in the context of NF-κB regulation and innate immunity. Using macrophages from TRIM21^−/−^ mice, the important role of TRIM21 in the cell response to opsonized bacteria and viruses has been demonstrated. TRIM21 catalyzes the formation of K63-linked ubiquitin chains and stimulates NF-κB, AP-1, IRF3, IRF5 and IRF7 pathways [57]. TRIM28 can participate in chromatin remodeling through its C-terminal bromo-domain, which recognizes acetylated lysine residues on histones [35]. The ability of TRIM28 to mitigate transcription can restrict replication of retroviruses, e.g., in embryonic stem cells [58]. We found that TRIM28 but not TRIM21 can contribute to NF-κB activation and IL-8 transcription in *H. pylori* infection. In addition, a supportive role of CDC37 in NF-κB and MAP kinase activation as well as in IL-8 transcription in *H. pylori* infection was identified. In agreement with this finding, inhibitors of CDC37/Hsp90, geldanamycin and witaferin A, have been found to possess anti-cancer properties by suppressing NF-κB signaling and the expression of pro-inflammatory mediators, integrins, laminins and extracellular matrix proteases [59–61]. In *H. pylori*-infected murine bone marrow-derived dendritic cells, withaferin A suppressed IκBα phosphorylation, expression of IL-1β and NLRP3, and cleavage of caspase-1 and IL-1β, which led to decreased IL-1β production [62]. Interestingly, CDC37/Hsp90 has been shown to associate with IKKα/IKKβ/NEMO, which is essential for the IKK complex maturation and stabilization as well as for TNF-induced activation of NF-κB in HeLa cells [59, 63]. Destruction of the CDC37/Hsp90/IKKs association and subsequent inhibition of the canonical and non-canonical NF-κB pathways can be used by hepatitis B virus as an immune evasion strategy in liver cells [64]. Previously, we have shown that in AGS cells TAK1 transiently associates with IKKα and IKKβ within 10–90 min p.i. with *H. pylori* [11]. Using MS, we could not detect IKKs within TAK1 associated proteins, but found that CDC37 was associated with TAK1. Presumably, CDC37 could serve as a linker between TAK1 and IKKs in *H. pylori*-infected cells.

Although we found no involvement of STOML2, the nucleic acid sensors PCBP1 and PCBP2, or of the ATP-dependent RNA helicase DDX3 in the regulation of NF-κB and MAP kinases by *H. pylori*, TNF and IL-1β, these proteins could play a role downstream of other stimuli, especially TGFβ and viruses. PCPB1 and PCPB2 have been shown to restrict cellular antiviral responses by linking Mitochondrial antiviral signaling (MAVS) to the E3 ligase ITCH, which leads to MAVS degradation [65, 66]. DDX3 is involved in RIG-I-mediated type I interferon production in response to viral infection [36]. Additionally, DDX3 is related to cell cycle, proliferation and apoptosis; thus, this protein is a possible prognostic marker and therapeutic target in oncology [67].

Although depletion of the TAK1-interacting proteins STOML2, PCPBs and DDX3 diminished TAK1 phosphorylation, the possibility that these proteins are downstream TAK1 substrates rather than upstream regulators cannot be excluded.

In conclusion, TAK1 resides in large protein complexes with TAB1, TAB2 and TAB3, phosphatases, STOML2, TRIM21 and TRIM28 in different cellular compartments. TAB1, TAB2, TRIM28 and CDC37 participate in TAK1 downstream NF-κB activation.

## MATERIALS AND METHODS

### Cell culture, bacteria and cytokines

AGS cells (ATCC^®^) were grown in Gibco^R^ RPMI 1640 medium (Life Technologies, Pasching, Austria) supplemented with 10% fetal bovine serum (FBS). Approximately 16 h prior to the experiment, the cell culture medium was replaced with fresh RPMI 1640 supplemented with 0.2% FBS. The CagA-expressing *H. pylori* strain P1 wild-type [11, 68] was cultured on GC agar supplemented with horse serum, vancomycin, trimethoprim, nystatin and vitamins in microaerophilic conditions for 48–72 h, and then a bacterial suspension in PBS was prepared. *H. pylori* was added to AGS cells at a multiplicity of infection of 100. Recombinant human TNFα and IL-1β (R&D Systems, Minneapolis, MN, USA) were used at a final concentration of 10 ng/ml.

### Antibodies

The description of the antibodies used for the experiments is provided in Supplementary Table 1.

### Cell fractionation, immunoprecipitation and immunoblotting

Cell lysates were prepared with a modified RIPA buffer (50 mM Tris-HCl, pH 7.5, 100 mM NaCl, 5 mM EDTA, 1% Triton X-100, 10% glycerol, 10 mM K_2_HPO_4_, 0.05% sodium deoxycholate, 1× protease inhibitor mixture (Roche Diagnostics GmbH, Mannheim, Germany), 1 mM Na_3_VO_4_, 1 mM Na_2_MoO_4_, 20 mM NaF, 0.1 mM PMSF, 20 mM β-glycerol-2-phosphate, 10 mM Na_4_P_2_O_7_). For analysis of TAK1 immunoprecipitates by MS, inhibitors of the ubiquitin and ubiquitin-like isopeptidases and deubiquitinylases N-ethylmaleimide (4 mM) (Sigma-Aldrich, Switzerland) and 1,10-phenantroline (2 mM) (Sigma-Aldrich, Austria) as well as the inhibitor of deacetylases nicotinamide (5 mM) and the inhibitor of OGA PUGNAc (50 μM) (Sigma-Aldrich, Saint Louis, Missouri, USA) were added to the lysis buffer. For immunoprecipitation, cell lysates were incubated with an antibody overnight at 4° C. Protein G Dynabeads^R^ (Invitrogen Dynal AS, Oslo, Norway) were used to capture the immunocomplexes. Subcellular fractions of AGS cells were prepared using a ProteoExtract kit (Calbiochem/Merck KGaA).

Samples were boiled in sample buffer (50 mM Tris-HCl, pH 6.8, 2% SDS, 10% glycerol, 3.3% ß-mercaptoethanol, and 0.1% bromophenol blue), subjected to SDS-PAGE and electrotransferred to Immobilon-P transfer polyvinylidene fluoride membranes (Millipore, Schwalbach, Germany). The blots were probed with the indicated primary antibodies diluted usually in 5% BSA. The HRP-conjugated goat secondary antibodies (heavy and light IgG chain specific or just light IgG chain specific; Jackson ImmunoResearch Laboratories Inc., PA, USA), Amersham^TM^ ECL^TM^ (GE Healthcare, Buckinghamshire, UK) and Pierce ECL Plus (Thermo Scientific, Rockford, IL, USA) substrates were used for antigen detection. The protein bands on X-ray films were scanned using a VersaDoc Imaging System (BioRad) and analyzed with Quantity One software (BioRad) using the global subtraction of background option. Densitometry values of bands of interest were all normalized relative to the respective GAPDH bands.

### Transfections

Unless stated otherwise, AGS cells (3.0 × 10^5^ cells/35 mm dish) were transfected with the following specific siRNAs: TAK1 (30 nM, Dharmacon^TM^/GE Healthcare, USA) or TAB1 (30 nM, Qiagen GmbH, Maryland, USA) for 48 h using siLentFect^TM^ Lipid Reagent (BioRad, Hercules, CA, USA); DDX3X (50 nM, Santa Cruz Biotechnology Inc.), STOML2 (40 nM, Dharmacon^TM^/GE Healthcare, USA), CDC37, PCBP1, PCBP2, TRIM21 or TRIM28 (50 nM, Dharmacon^TM^/GE Healthcare) using Attractene^®^ transfection reagent (Qiagen GmbH, Hilden, Germany) for 72 h in cell culture medium supplemented with 10% FCS. Scrambled sequences, which should not lead to the destruction of any known cellular mRNA, were purchased from Santa Cruz Biotechnology Inc. and Dharmacon^TM^. TAB2 knock-out cells were selected from AGS cells transfected with TAB2 CRISPR/Cas9 KO and HDR plasmids (2 μg of each, Santa Cruz Biotechnology Inc.) using puromycin. For transient TAK1 and/or TAB1 overexpression, AGS cells (2 × 10^6^ cells/100 mm dish) were transfected with pCMV-TAK1 and/or pCMV-TAB1 (provided by K. Matsumoto) using Attractene^®^ transfection reagent (Qiagen GmbH).

### Transactivation assay

AGS cells were seeded onto 24-well plates at a density of 0.6 × 10^5^ cells per well in RPMI1640 culture medium supplemented with 10% FCS. Firefly luciferase plasmid containing five copies of an NF-κB response element (Promega, Madison, WI, USA) was mixed with *Renilla* Luciferase plasmid at a ratio of 50:1 and transfected using Attractene^®^ transfection reagent (Qiagen GmbH). siRNAs were co-transfected. The luciferase activity was estimated in cell lysates 48 h post transfection and 3.5 h post stimulation using a Dual-Luciferase Reporter Assay System (Promega) with a Lumat LB 9507 luminometer (Berthold Technologies, Bad Wildbad, Germany). The inducible firefly luciferase activity was normalized relative to *Renilla* luciferase activity, and fold changes in stimulated samples were calculated compared to non-stimulated cells.

### RNA isolation and RT-PCR

Total RNA was extracted using an RNeasy^®^Plus Micro kit (Qiagen). cDNA was synthesized from 1 μg of RNA using a random hexamer primer and a RevertAid^TM^ First Strand cDNA Synthesis kit (Thermo Scientific, Lithuania, EU). Then, 1 μl of cDNA mixture was combined with primers (0.25 μM), fluorescein (1:10^5^; BioRad) and components of SensiMix SYBR Mastermix (Bioline Reagents Ltd., United Kingdom) and amplified in an iCycler IQ (BioRad). The following primers were used: 5ʹ-AGATGTCAGTGCATAAAGACA-3ʹ (forward) and 5ʹ-TATGAATTCTCAGCCCTCTTCAAAAA-3ʹ (reverse) for IL-8; 5ʹ-TCCAAAATCAAGTGGGGCGATGCT-3ʹ (forward) and 5ʹ-CCACCTGGTGCTCAGTGTAGCCC-3ʹ (reverse) for GAPDH. GAPDH expression served as an internal control. Serial dilutions of the dipeptidyl peptidase IV gene cloned into a pCRR2.1-TOPO vector and the primers 5ʹ-GATGCTACAGCTGACAGTCGC-3ʹ (forward) and 5ʹ-TGGTGACCATGTGACCCACTG-3ʹ (reverse) served for generation of a calibration curve.

### Mass spectrometry

Sample preparation for MS was performed via onBeads digestion for the best analytical sensitivity. In brief, beads were rehydrated in 50 mM NH_4_HCO_3_, pH 8.0, and subsequently incubated with 1 mM DTT at 56° C for 45 min. Afterwards, reduced cysteine residues were ß-methylthiolated via the addition of 5 mM methyl methanethiosulfonate at room temperature for 30 min. Proteins were digested by adding 0.5 μg trypsin (Trypsin Gold, Promega) and incubating at 37° C overnight. The generated tryptic peptides were gathered by collecting the supernatants combined with two washing steps of the beads using 50 μl of 25 mM NH_4_HCO_3_ for each wash. All supernatants of a sample were pooled and dried down in a vacuum centrifuge. The peptides were redissolved in 5 μl 0.1% trifluoroacetic acid (TFA) and purified on ZIP-TIP, C18-nanocolumns (Millipore, Billerica, USA). The peptides were eluted in 7 μl of 70% (v/v) ACN and subsequently dried in a vacuum centrifuge.

LC-MS/MS was performed on a hybrid dual-pressure linear ion trap/orbitrap mass spectrometer (LTQ Orbitrap Velos Pro, Thermo Scientific, San Jose, CA) equipped with an EASY-nLC Ultra HPLC (Thermo Scientific, San Jose, CA). The peptide samples were dissolved in 10 μl of 2% ACN/0.1% TFA and fractionated on a 75 μm I.D., 25 cm PepMap C18-column, packed with 2 μm resin (Dionex, Germany). Separation was achieved by applying a gradient from 2% ACN to 35% ACN in 0.1% FA over a 150 min gradient at a flow rate of 300 nl/min.

The LTQ Orbitrap Velos Pro MS exclusively used CID-fragmentation when acquiring MS/MS spectra consisted of an orbitrap full MS scan followed by up to 10 LTQ MS/MS experiments (TOP10) on the most abundant ions detected in the full MS scan. The essential MS settings were as follows: full MS (FTMS; resolution 60.000; m/z range 400–2000); MS/MS (Linear Trap; minimum signal threshold 500; isolation width 2 Da; dynamic exclusion time setting 30 s; singly charged ions were excluded from selection). Normalized collision energy was set to 35%, and the activation time was set to 10 ms.

Raw data processing, protein identification and PTMs assignment of the high resolution orbitrap data sets was performed with the *de novo* sequencing algorithms of PEAKS Studio 7.0 (Bioinformatics Solutions) and ProteomeDiscoverer 1.4 (Thermo Scientific). The false discovery rate was set to <1%.

Immunoprecipitation with isogenic antibody (Cell Signaling Technology) was performed in each experiment to filter TAK1-unspecific binding proteins during data analysis. Three (two for mock-transfected cells) independent experiments for each experimental condition were performed and analyzed. Proteins that were found in at least 2 experiments were further considered as true interactors.

The MS data obtained in this study are deposited to the ProteomeXchange Consortium [69] via the PRIDE partner repository with the dataset identifier PXD007632.

### Network visualization

The result files for each experimental condition (bait) were processed using a simple Python script and imported into Cytoscape [70] version 3.0, where they were consolidated into a single bait-prey network (i.e., without prey redundancy) using Cytoscape's default network merge tool. This bait-prey network was then exported to a graphml network format to allow import into the Gephi network analysis platform [71] for final layout (*forcedirected2*), style editing and visualization. Two databases, http://www.ebi.ac.uk/intact/ and http://www.innatedb.com/, were used to find already known human TAK1 interacting proteins and compare them with our TAK1 interactome. Only experimentally validated direct protein-protein interactions, which are annotated to the imex standard (http://www.imexconsortium.org/), were taken into consideration.

### Statistical analysis

Except MS data processing, significance was calculated using two-tailed unpaired Student's *t*-test. The data are presented as the mean of at least 3 separate experiments ± SEM with the value of the control arbitrarily normalized to 1.

## SUPPLEMENTARY MATERIALS FIGURES AND TABLES





## Abbreviations

CCT: chaperoning T complex
EGFR: epidermal growth factor receptor
FBS: fetal bovine serum
GF: growth factor
HSPs: heat shock proteins
IKKs: IκB kinases
MAP kinase: mitogen-activated protein kinase
MAPK8IP1: MAP kinase 8 interacting Protein 1
MAVS: mitochondrial antiviral signaling
MS: mass spectrometry
NF-κB: nuclear factor κB
PCBP: poly(rC)-binding protein
PP: protein phosphatase
PTMs: post-translational modifications
STOML2: stomatin-like protein 2
TAB: TAK1-binding protein
TGFβ: transforming growth factor-β
TAK1: TGFβ-activated kinase 1
TFA: trifluoroacetic acid
TLRs: toll-like receptors
TRIM: tripartite motif
T4SS: type 4 secretion system
