# Supplementary material for: Interactome analysis of transforming growth factor-β-activated kinase 1 in Helicobacter pylori-infected cells revealed novel regulators tripartite motif 28 and CDC37
Source: Oncotarget. 2018 Feb 21;9(18):14366–81. doi: 10.18632/oncotarget.24544 (PMC5865676; doi:10.18632/oncotarget.24544)
Supplement: Supplementary file 1 [file oncotarget-09-14366-s001.pdf]

Interactome analysis of transforming growth factor-β-activated kinase 1 in *Helicobacter pylori*-infected cells revealed novel regulators tripartite motif 28 and CDC37

SUPPLEMENTARY MATERIALS

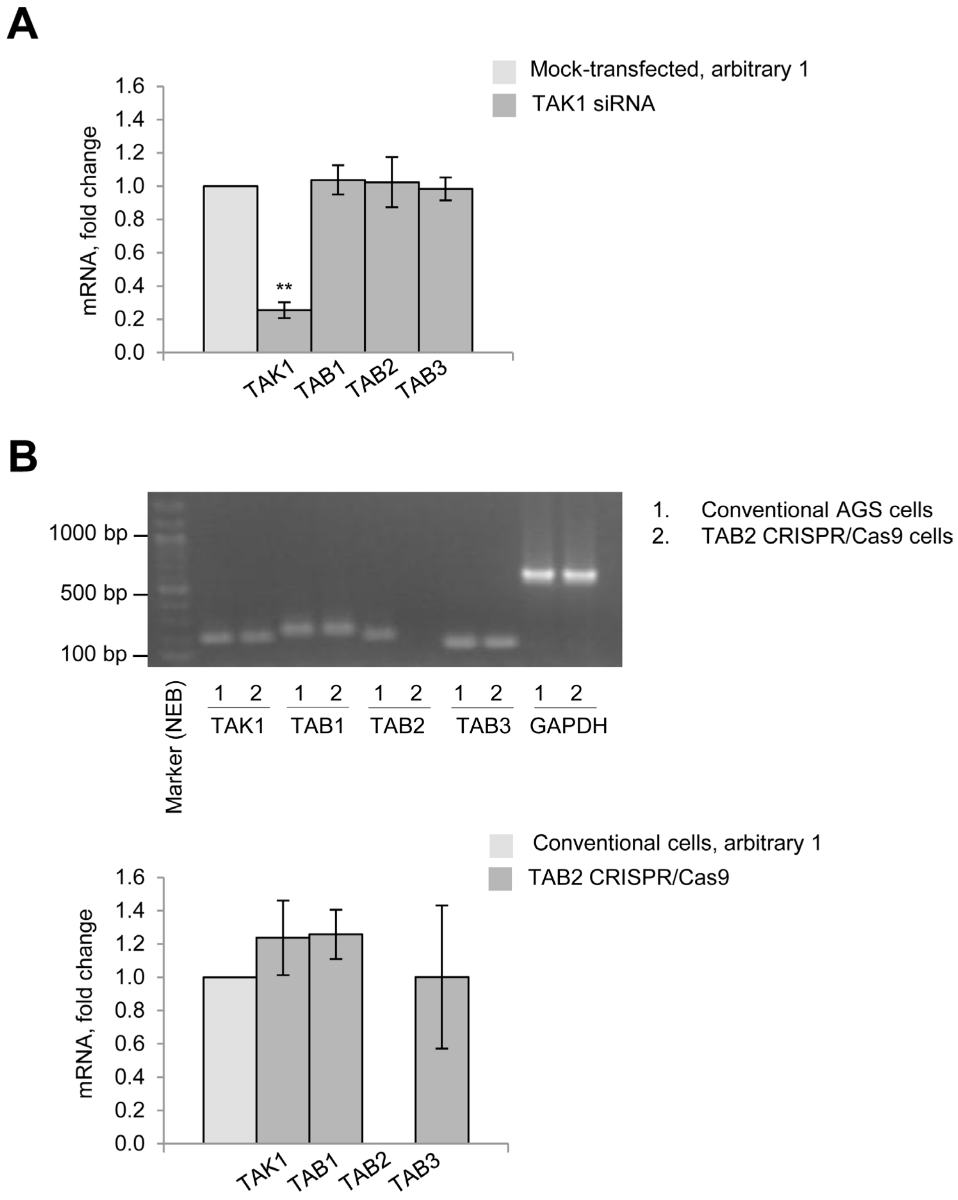

Supplementary Figure 1: Depletion of TAK1 (**A**) and TAB2 (**B**) does not affect mRNAs of other members of TAK1/TABs complex. Total RNA was isolated from mock- or TAK1 siRNA-trasfected cells and from TAB2 CRISPR-Cas9 AGS cell clone, and qRT-PCRs were performed. In addition to described in the Materials and Methods, the following primers (Invitrogen/Thermo Fisher Scientific) were used: 5′-ACAGTGTTCCCAAGGAGTGG-3′ (forward) and 5′-AACTTCAGGTGCCATCCAAG-3′ (reverse) for TAK1; 5′-GGATCGGGGATTACAAGGTT-3′ (forward) and 5′-GCTTGGCAAACTCAGTGTCA-3′ (reverse) for TAB1; 5′-TCAACAGCCAAATCAGCAAG-3′ (forward) and 5′-TTTCTTTGTGGGGGTTCAAG-3′ (reverse) for TAB2; 5′-ACAGCAGCAGATCCCTCAGT-3′ (forward) and 5′-TGATATGGATGGGGTGGAGT-3′ (reverse) for TAB3. $^{**}p < 0.01$ vs mock-transfected cells.

A

Conditions		TAK1 phosphorylation		
Overexpression	Stimuli	Exp. 1	Exp. 2	Exp.3
mock	-	T415	S389<br>S417<br>S439<br>S455	
TAK1	-	S389<br>S417<br>S439<br>S455	S389<br>S417<br>S439<br>T444<br>S455	S389<br>S439<br>T444<br>S455
TAK1	*H. pylori*, 20 min	S389<br>S439<br>S455	S389<br>S439<br>S455	S389<br>S417<br>S439<br>T444<br>S455
TAK1	*H. pylori*, 45 min	S389<br>T415<br>S439<br>T444<br>S455	S439<br>S455	S389<br>S417<br>S439<br>S455
TAK1, TAB1	-	S375<br>S389<br>S439<br>T444<br>S455	S361<br>S389<br>T415<br>S439<br>T444<br>S454<br>S455	S305<br>T344<br>S389<br>T415<br>S439<br>S446<br>S448<br>S454<br>S455<br>T489<br>S492
TAB1	-	S417<br>S439<br>T444<br>S454	S439<br>S455	S417<br>S439

B

Conditions		TAK1 acetylation		
Overexpression	Stimuli	Exp. 1	Exp. 2	Exp.3
mock	-	no pep.	no pep.	
TAK1	-	no pep.	M18	no pep.
TAK1	*H. pylori*, 20 min	no pep.	S2	M18
TAK1	*H. pylori*, 45 min	no pep.	Q359	no pep.
TAK1, TAB1	-	M18	M18	M18<br>K547
TAB1	-	no pep.	no pep.	no pep.

Supplementary Figure 2: Phosporylated (**A**) and acetylated (**B**) residues identified with MS in endogenous ("mock" and "TAB1") and overexpressed TAK1 ("TAK1" and "TAK1, TAB1").

A

Conditions		TAB1 phosphorylation			TAB1 HexNAcylation		
Overexpression	Stimuli	Exp. 1	Exp. 2	Exp.3	Exp. 1	Exp. 2	Exp.3
mock	-	-	S7		S395	S396	
TAK1	-	no pep.	S11	S7	S396	S401	S401
TAK1	*H. pylori*, 20 min	S11	S7	no pep.	S395	S401	S401
TAK1	*H. pylori*, 45 min	S7		-	S396		S396
TAK1, TAB1	-	S7	S11<br>S16<br>S378<br>S469	S7<br>S11<br>T18<br>S378	S395<br>S401	S401	S395<br>S401
TAB1	-	S7	S7	S11	S396	S401	S401

B

Conditions		TAB2 phosphorylation			TAB2 acetylation		
Overexpression	Stimuli	Exp. 1	Exp. 2	Exp.3	Exp. 1	Exp. 2	Exp.3
mock	-	no pep.	no pep.		A2	A2	
TAK1	-	no pep.	no pep.	no pep.	A2	A2	A2
TAK1	*H. pylori*, 20 min	T484	S477<br>T488	S482	A2	A2	no pep.
TAK1	*H. pylori*, 45 min	no pep.	no pep.	no pep.	A2	A2	no pep.
TAK1, TAB1	-	no pep.	no pep.	no pep.	no pep.	no pep.	no pep.
TAB1	-	no pep.	no pep.	S482<br>T660	A2	A2	A2

C

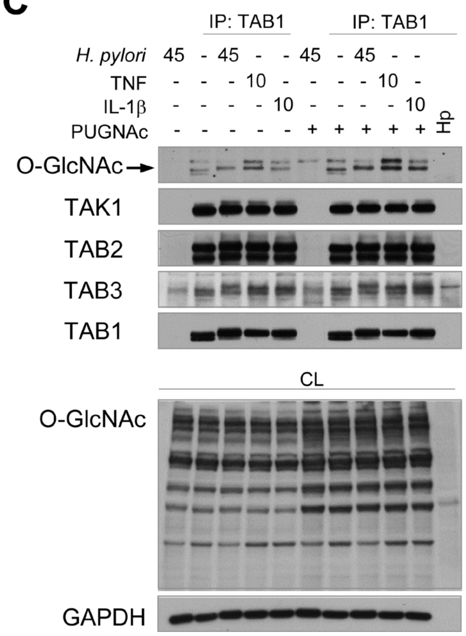

Supplementary Figure 3: Phosporylated, glycosylated and acetylated residues identified with MS in TAB1 (**A**) and TAB2 (**B**) co-immunoprecipitated with TAK1. (**C**) TAB1 is target for glycosylation. AGS cells were left untreated or were incubated with PUGNAc overnight. Next, they were treated with *H. pylori*, TNF or IL-1β for the indicated times (in min), cell lysates (CL) and TAB1 immunoprecipitates (IP) were prepared and analyzed by immunoblotting. *H. pylori* lysate (Hp) was used to demonstrate no cross-reactivity of the antibodies. In some lysates, the immunoprecipitation antibody was not added to approve non-specific protein binding to the beads (lanes 1 and 6). “no pep.”, no peptide with the PTM was detected.

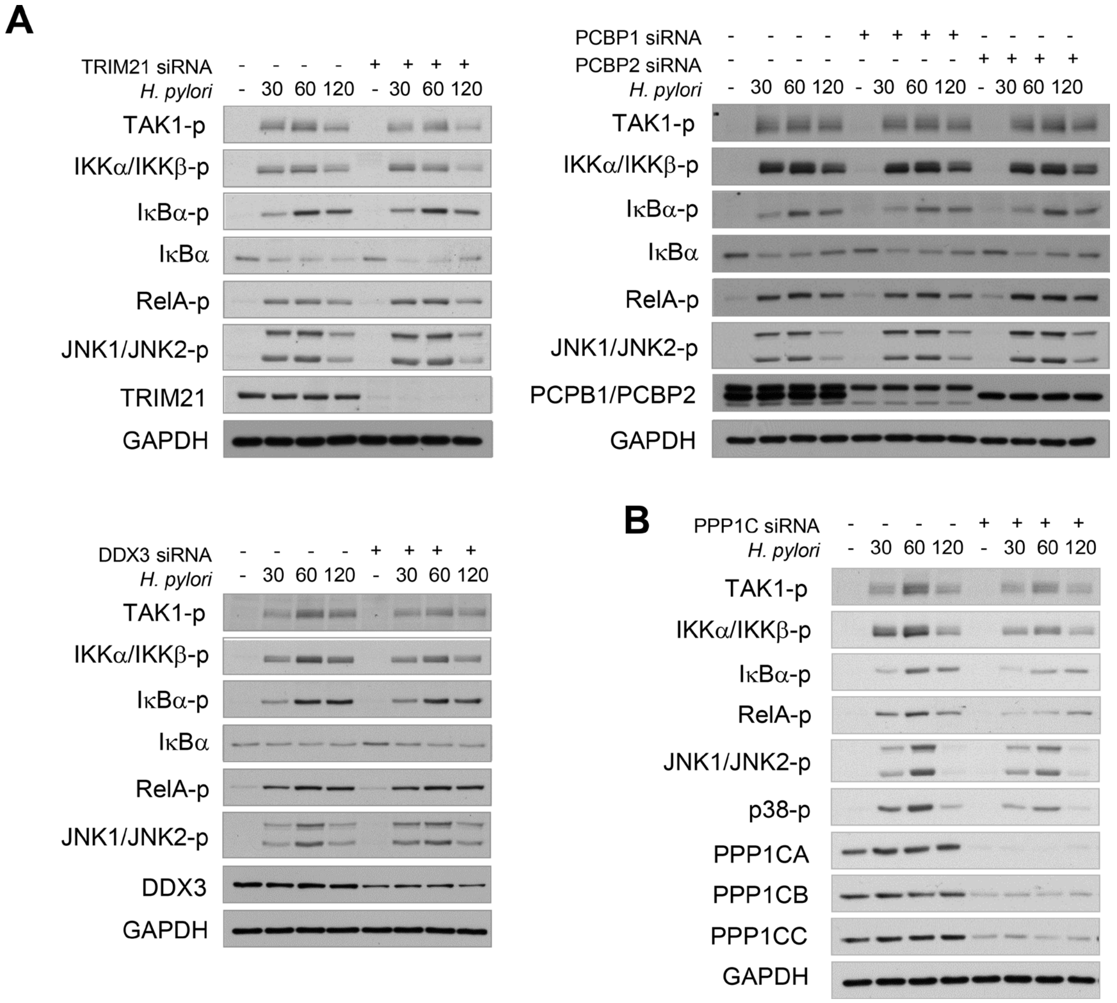

Supplementary Figure 4: (**A**) TRIM21, PCBPs and DDX3X are not required for NF-κB and MAP kinases activation in *H. pylori*-infected AGS cells. **(B**) Concomitant depletion of PPP1CA, PPP1CB and PPP1CC leads to decrease in posphorylation of NF-κB and MAP kinases in *H. pylori*-infected AGS cells. Mock- or specific siRNA-trasfected cells were treated with *H. pylori* for the indicated times (in min), cell lysates were prepared and analysed by immunoblotting.

Supplementary Images: Uncropped immunoblots. See Supplementary Images

Supplementary Table 1: Antibodies used for the investigation

Name	Sourse	Supplier	Mr of target, kD
CDC37	rb	Bethyl Laboratiries Inc./Biomol, Hamburg, Germany	50
DDX3	mouse	Abcam	73
GAPDH	mouse	Millipore, Temecula, CA, USA	36
Histone H3	rb	Cell Signalling Technology Inc., Danvers, MA, USA	17
IκBα (44D4)	rb	Cell Signalling Technology Inc., Danvers, MA, USA	40
JNK1, JNK2	rb	Sigma, St. Louis, MO, USA	46, 54
NF-κB p65	mouse	BD Biosciences Pharmingen, San Jose, CA, USA	65
Occludin	mouse	BD Biosciences Pharmingen, San Jose, CA, USA	65
PCBP2	rb	LCBio/ Biozol, Eching, Germany	37, 5 (PCBP1), 38, 6 (PCBP2)
Phospho-ATF-2 (Thr71)	rb	Cell Signalling Technology Inc., Danvers, MA, USA	70
Phospho-IκBα (Ser32/36) (5A5)	mouse	Cell Signalling Technology Inc., Danvers, MA, USA	40
Phospho-IKKα/β (Ser176/177) (C84E11)	rb	Cell Signalling Technology Inc., Danvers, MA, USA	85 (IKKα), 87 (IKKβ)
Phospho-NF-κB p65 (Ser536)	rb	Cell Signalling Technology Inc., Danvers, MA, USA	65
Phospho-p38 (Thr180/Tyr182)	mouse	Sigma, St. Louis, MO, USA	38
Phospho-SAPK/JNK (T183/ Y185)	rb mono	Cell Signalling Technology Inc., Danvers, MA, USA	46, 54
Phospho-TAK1 (Thr184/187)	rb	Cell Signalling Technology Inc., Danvers, MA, USA	78 to 82
PPP1CA	rb	Bethyl Laboratiries Inc./Biomol, Hamburg, Germany	37
PPP1CB	rb	Bethyl Laboratiries Inc./Biomol, Hamburg, Germany	37
PPP1CC	rb	Bethyl Laboratiries Inc./Biomol, Hamburg, Germany	37
STOML2 (siRNA depletion experiments)	rb	Abgent, San Diego, CA, USA/Biomol GmbH, Hamburg, Germany	38,5
STOML2 (co-precipated with TAK1)	rb	LCBio/ Biozol, Eching, Germany	38,5
TAK1 (D94D7) (for IP), against residues surrounding Gln600	rb mono	Cell Signalling Technology Inc., Danvers, MA, USA	78 to 82
TAK1 (for WB)	rb	Cell Signalling Technology Inc., Danvers, MA, USA	78 to 82
TAB1 (C25E9)	rb	Cell Signalling Technology Inc., Danvers, MA, USA	60
TAB2, against region between residues 500 and 550	rb	Novus Biologicals, Abingdon, United Kingdom	76 to 80
TAB2 (C88H10), against residues surrounding Leu330	rb mono	Cell Signalling Technology Inc., Danvers, MA, USA	76 to 80
TAB3	rb	Acris GmbH, Herford, Germany	79 to 83
TRIM21 (SSA1)	rb	Bethyl Laboratiries Inc./Biomol, Hamburg, Germany	50
TRIM28 (KAP-1)	rb	Bethyl Laboratiries Inc./Biomol, Hamburg, Germany	100
