# Supplementary material for: Interactome analysis of transforming growth factor-β-activated kinase 1 in Helicobacter pylori-infected cells revealed novel regulators tripartite motif 28 and CDC37
Source: Oncotarget. 2018 Feb 21;9(18):14366–81. doi: 10.18632/oncotarget.24544 (PMC5865676; doi:10.18632/oncotarget.24544)
Supplement: Supplementary file 2 [file oncotarget-09-14366-s002.pdf]

To Figure 1

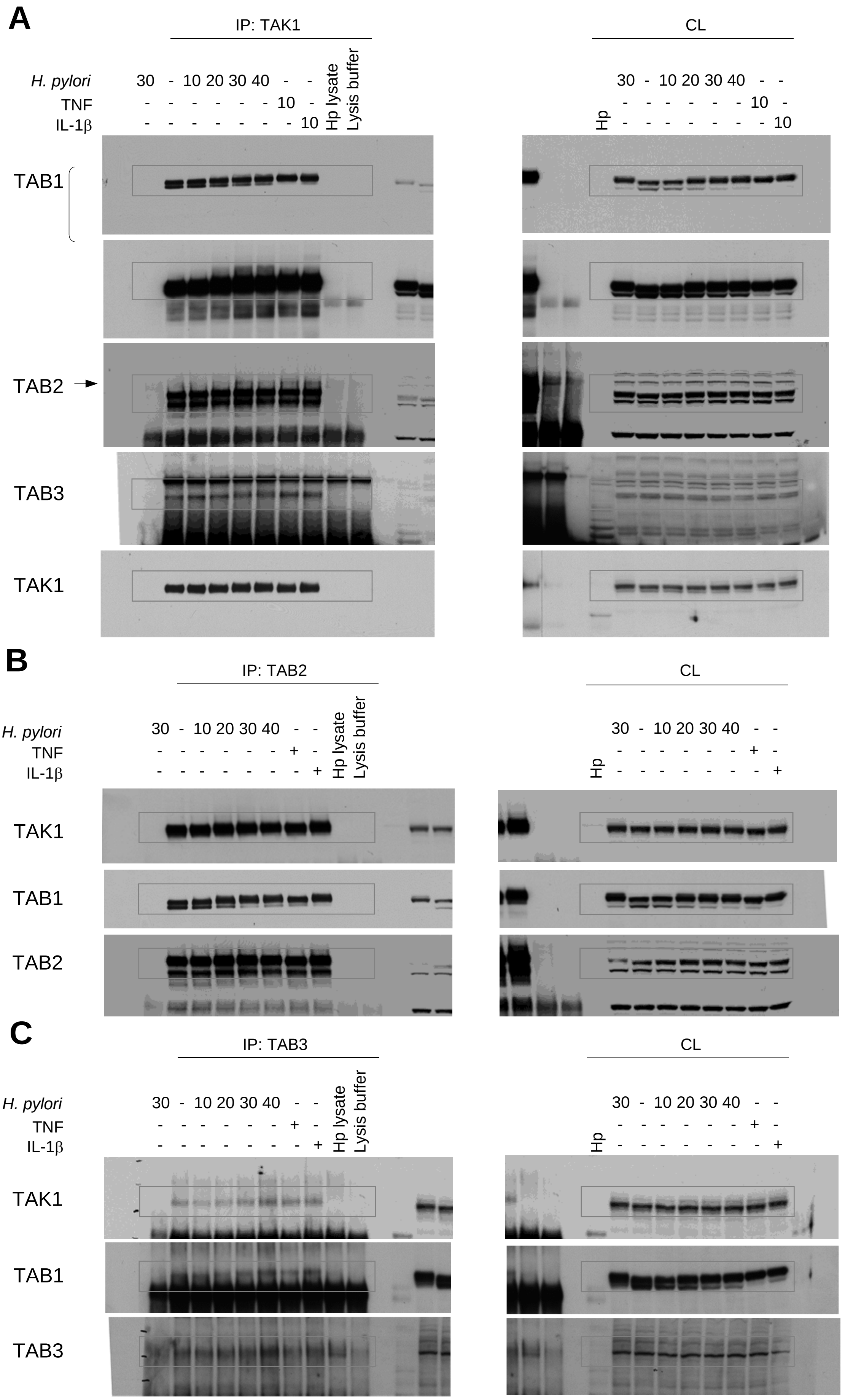

A
IP: TAK1
CL
H. pylori
30 - 10 20 30 40 - -
TNF
- - - - - - - 10 -
IL-1β
- - - - - - - - 10
Hp lysate
Lysis buffer
Hp
TAB1
TAB2
TAB3
TAK1
B
IP: TAB2
CL
H. pylori
30 - 10 20 30 40 - -
TNF
- - - - - - - + -
IL-1β
- - - - - - - - +
Hp lysate
Lysis buffer
Hp
TAK1
TAB1
TAB2
C
IP: TAB3
CL
H. pylori
30 - 10 20 30 40 - -
TNF
- - - - - - - + -
IL-1β
- - - - - - - - +
Hp lysate
Lysis buffer
Hp
TAK1
TAB1
TAB3

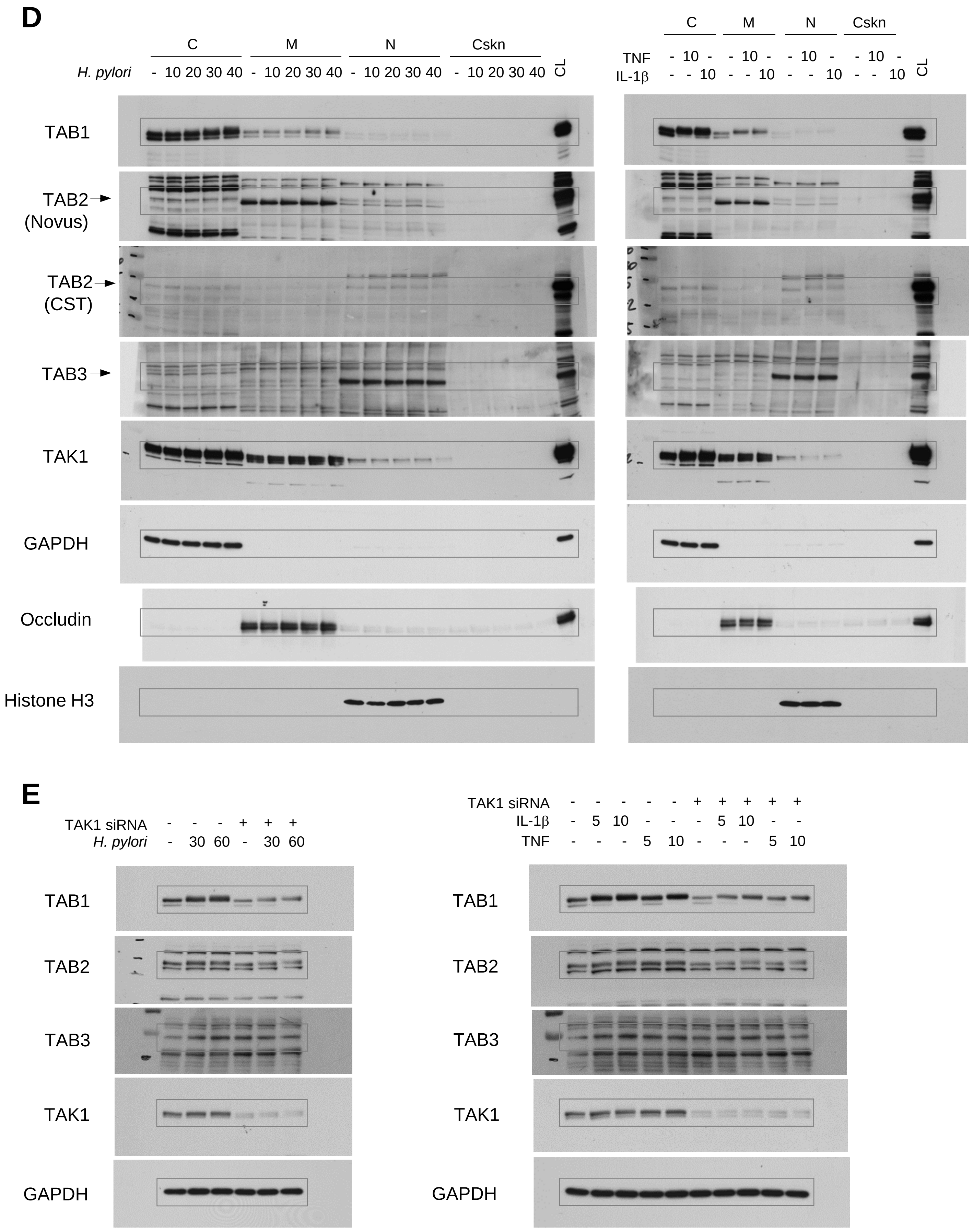

D
C
M
N
Cskn
H. pylori
- 10 20 30 40 - 10 20 30 40 - 10 20 30 40 - 10 20 30 40
CL
TNF
- 10 - - 10 - - 10 - - 10 -
IL-1β
- - 10 - - 10 - - 10 - - 10
TAB1
TAB2 (Novus)
TAB2 (CST)
TAB3
TAK1
GAPDH
Occludin
Histone H3
E
TAK1 siRNA
- - - + + +
H. pylori
- 30 60 - 30 60
TAK1 siRNA
- - - - - + + + + +
IL-1β
- 5 10 - - - 5 10 - -
TNF
- - - 5 10 - - - 5 10
TAB1
TAB2
TAB3
TAK1
GAPDH

To Figure 2

A

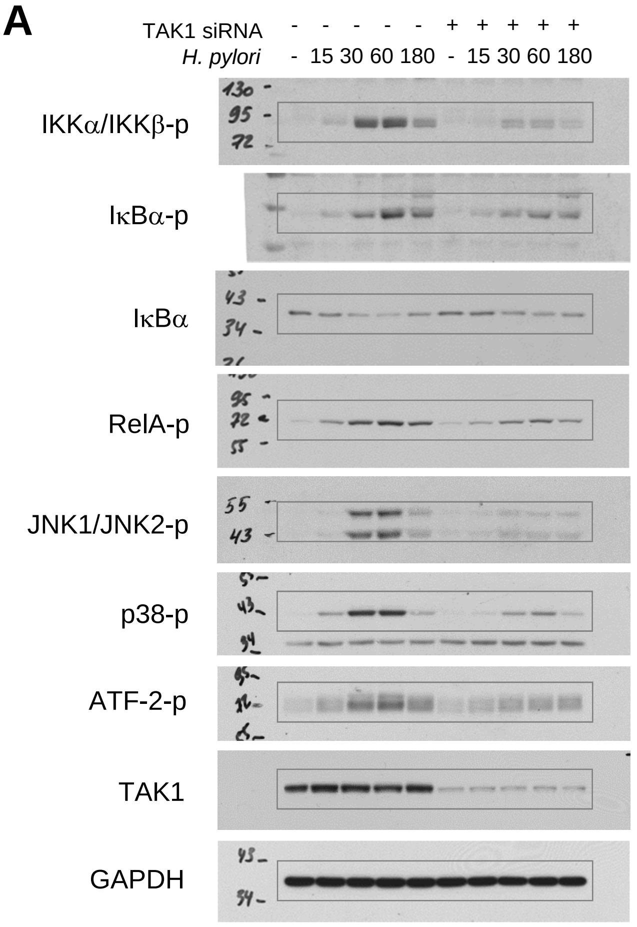
TAK1 siRNA - - - - - + + + + +
H. pylori - 15 30 60 180 - 15 30 60 180
IKKα/IKKβ-p
IκBα-p
IκBα
RelA-p
JNK1/JNK2-p
p38-p
ATF-2-p
TAK1
GAPDH

B

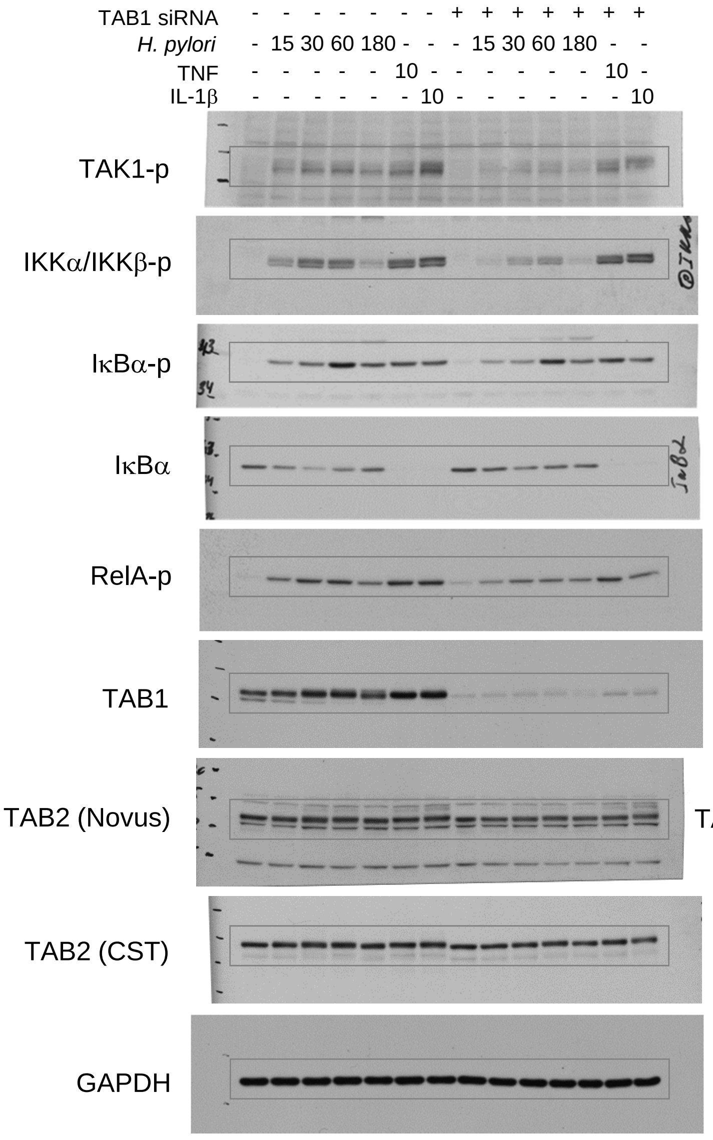
TAB1 siRNA - - - - - - - + + + + + + +
H. pylori - 15 30 60 180 - - - 15 30 60 180 - -
TNF - - - - - 10 - - - - - - 10 -
IL-1β - - - - - - 10 - - - - - - 10
TAK1-p
IKKα/IKKβ-p
IκBα-p
IκBα
RelA-p
TAB1
TAB2 (Novus)
TAB2 (CST)
GAPDH

C

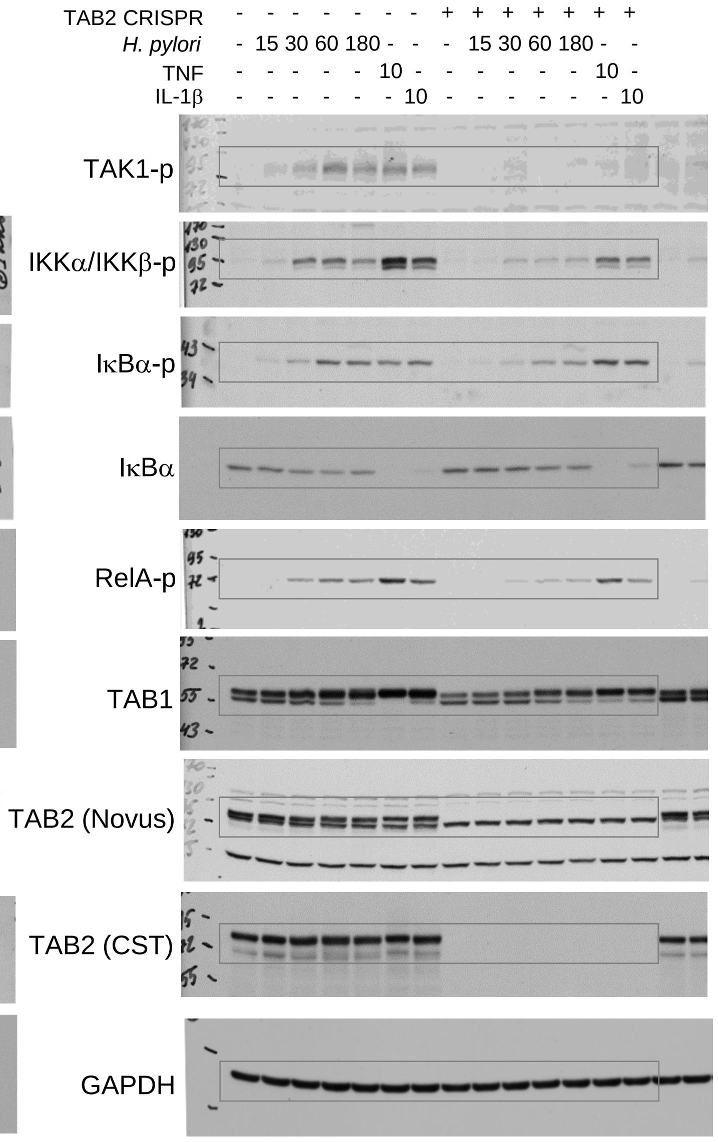
TAB2 CRISPR - - - - - - - + + + + + + +
H. pylori - 15 30 60 180 - - - 15 30 60 180 - -
TNF - - - - - 10 - - - - - - 10 -
IL-1β - - - - - - 10 - - - - - - 10
TAK1-p
IKKα/IKKβ-p
IκBα-p
IκBα
RelA-p
TAB1
TAB2 (Novus)
TAB2 (CST)
GAPDH

To Figure 3

A

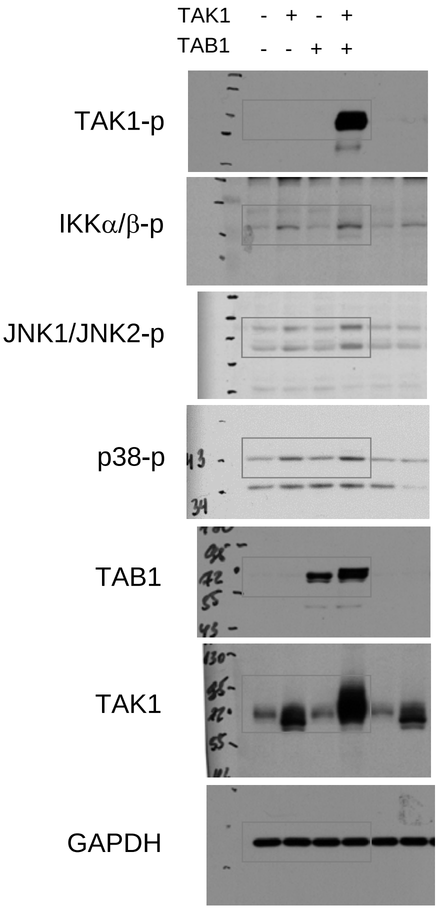

To Figure 4

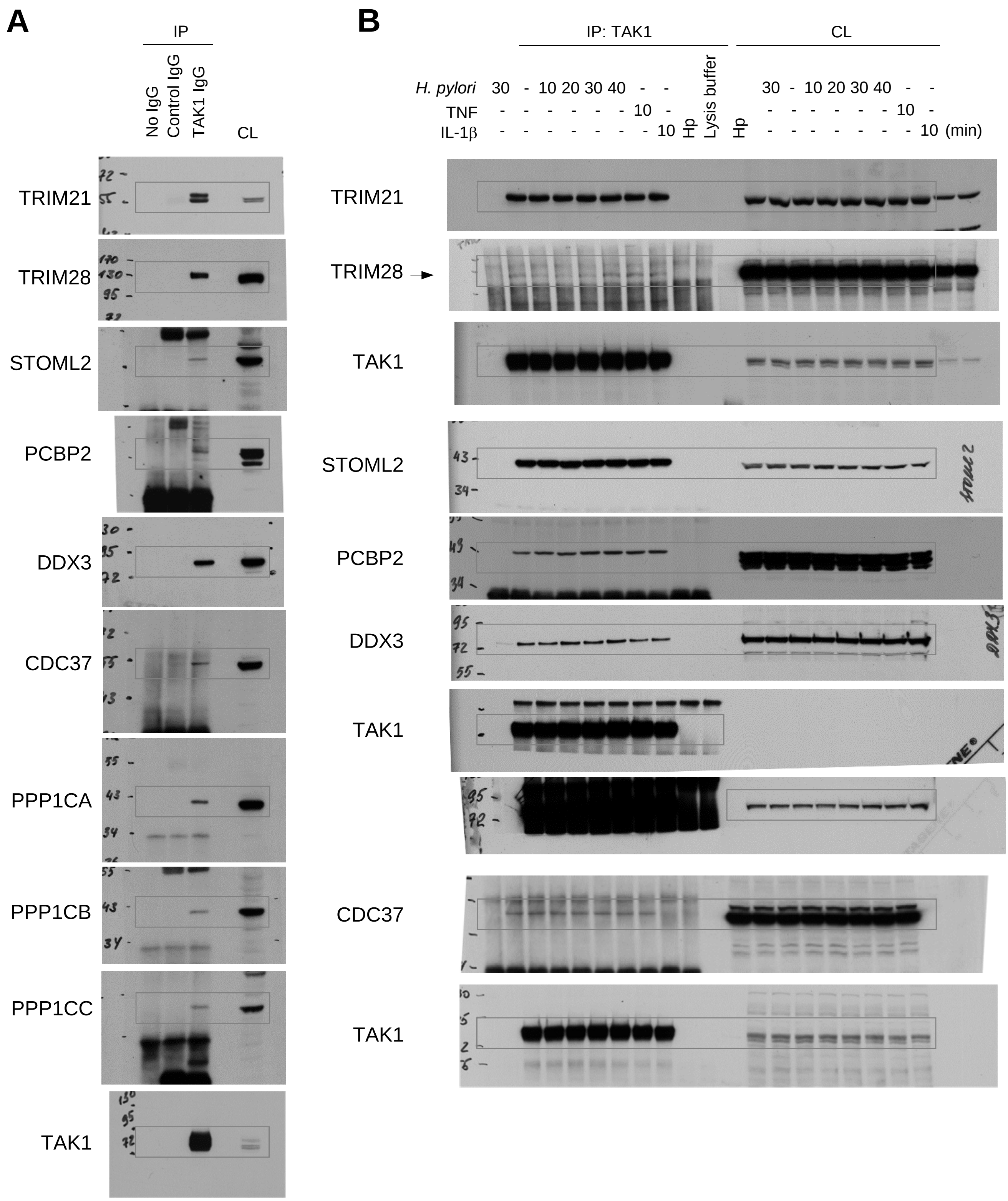
A
IP
No IgG
Control IgG
TAK1 IgG
CL
TRIM21
TRIM28
STOML2
PCBP2
DDX3
CDC37
PPP1CA
PPP1CB
PPP1CC
TAK1
B
IP: TAK1
CL
H. pylori 30 - 10 20 30 40 - -
TNF - - - - - - - 10 -
IL-1β - - - - - - - - 10
Hp
Lysis buffer
Hp
30 - 10 20 30 40 - -
- - - - - - - 10 -
- - - - - - - - 10 (min)
TRIM21
TRIM28
TAK1
STOML2
PCBP2
DDX3
TAK1
CDC37
TAK1

C

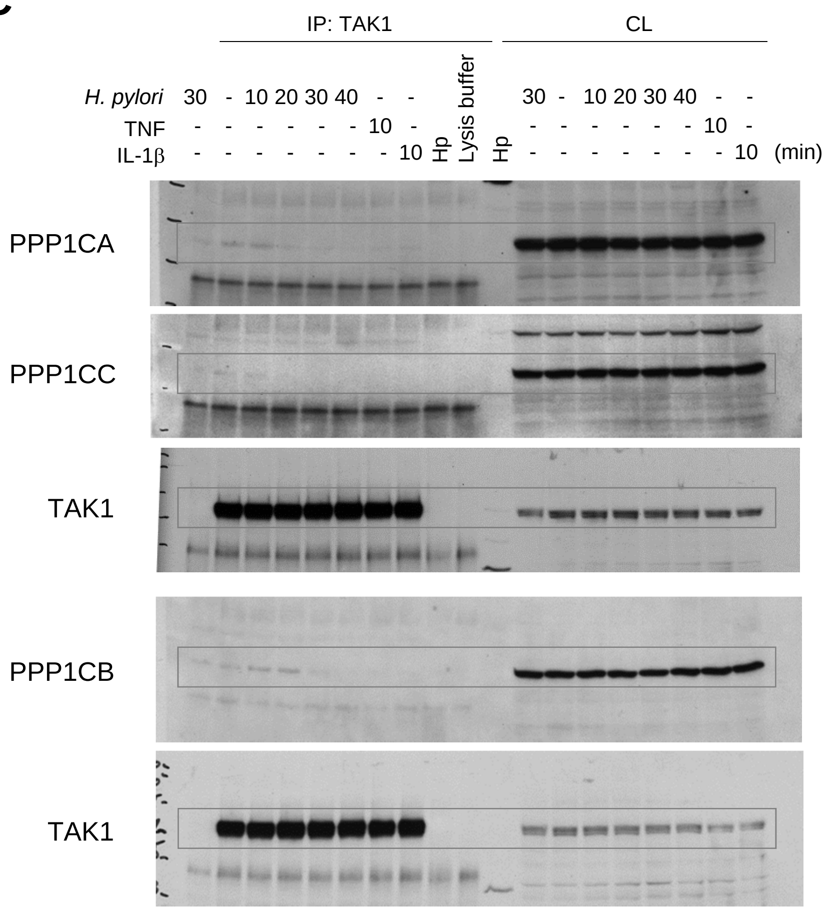
IP: TAK1
CL
H. pylori 30 - 10 20 30 40 - - 30 - 10 20 30 40 - -
TNF - - - - - - 10 - - - - - - - 10 -
IL-1β - - - - - - - 10 Hp Lysis buffer Hp - - - - - - - 10 (min)
PPP1CA
PPP1CC
TAK1
PPP1CB
TAK1

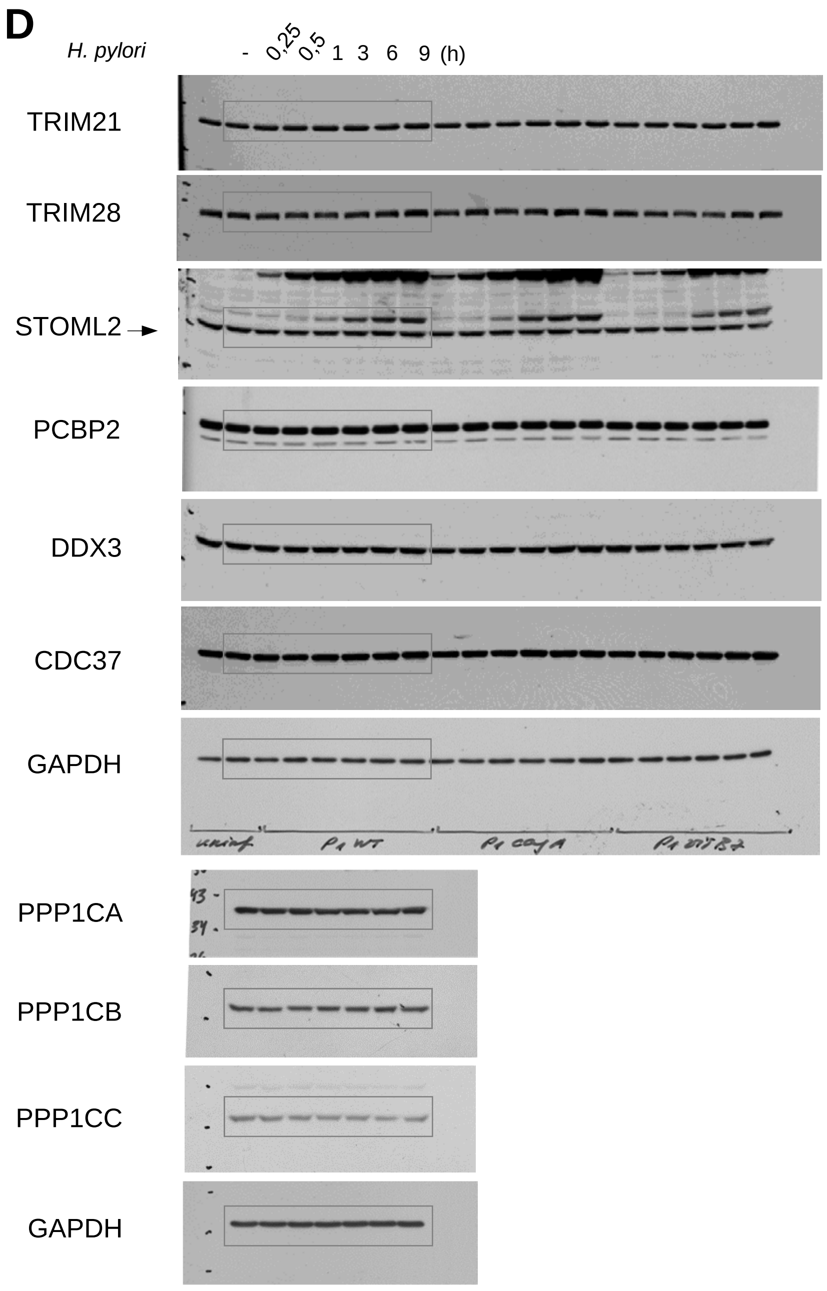
D
H. pylori
- 0,25 0,5 1 3 6 9 (h)
TRIM21
TRIM28
STOML2
PCBP2
DDX3
CDC37
GAPDH
PPP1CA
PPP1CB
PPP1CC
GAPDH

To Figure 5

A

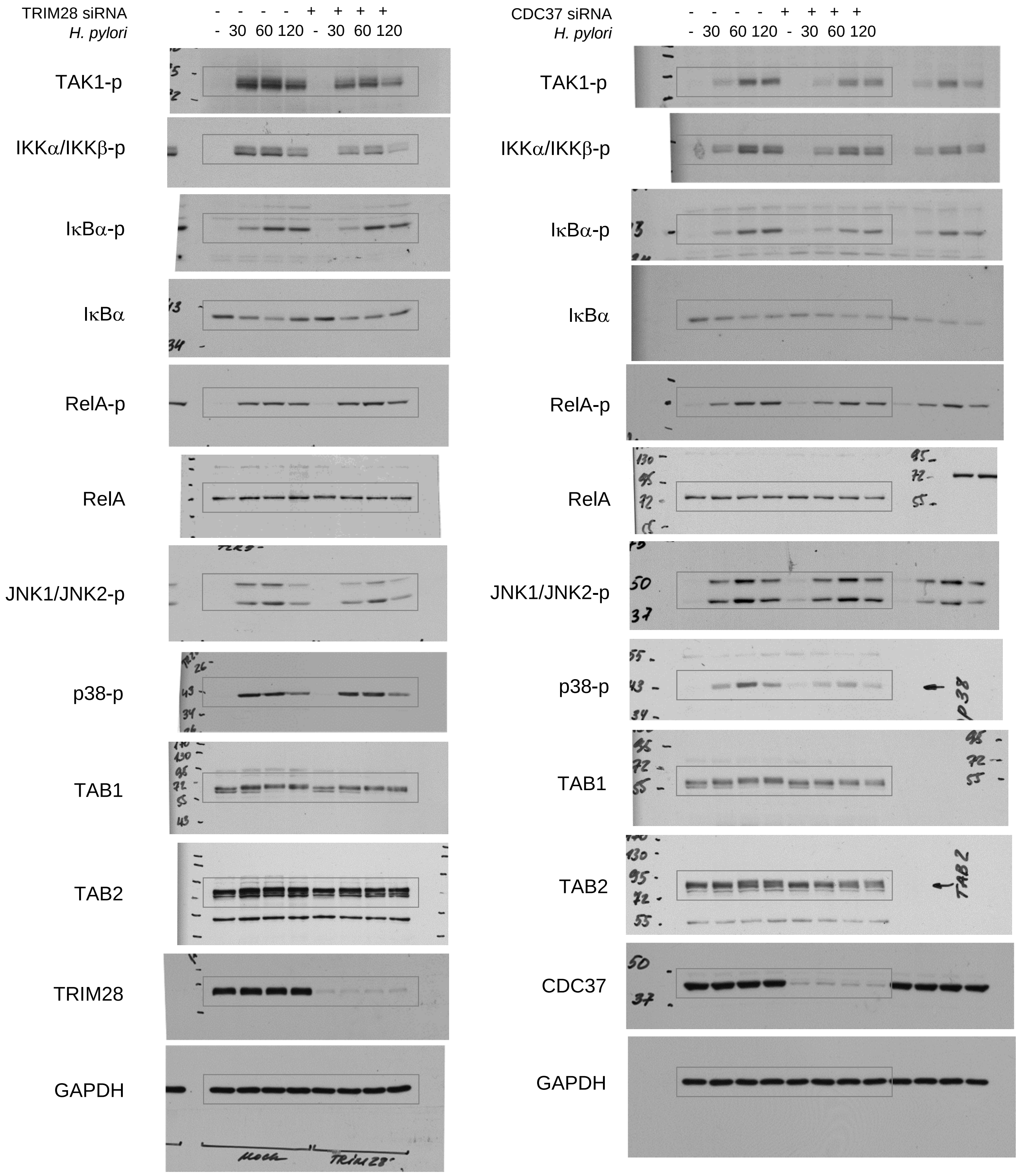

TRIM28 siRNA - - - - + + + +
H. pylori - 30 60 120 - 30 60 120
TAK1-p
IKKα/IKKβ-p
IκBα-p
IκBα
RelA-p
RelA
JNK1/JNK2-p
p38-p
TAB1
TAB2
TRIM28
GAPDH
CDC37 siRNA - - - - + + + +
H. pylori - 30 60 120 - 30 60 120
TAK1-p
IKKα/IKKβ-p
IκBα-p
IκBα
RelA-p
RelA
JNK1/JNK2-p
p38-p
TAB1
TAB2
CDC37
GAPDH

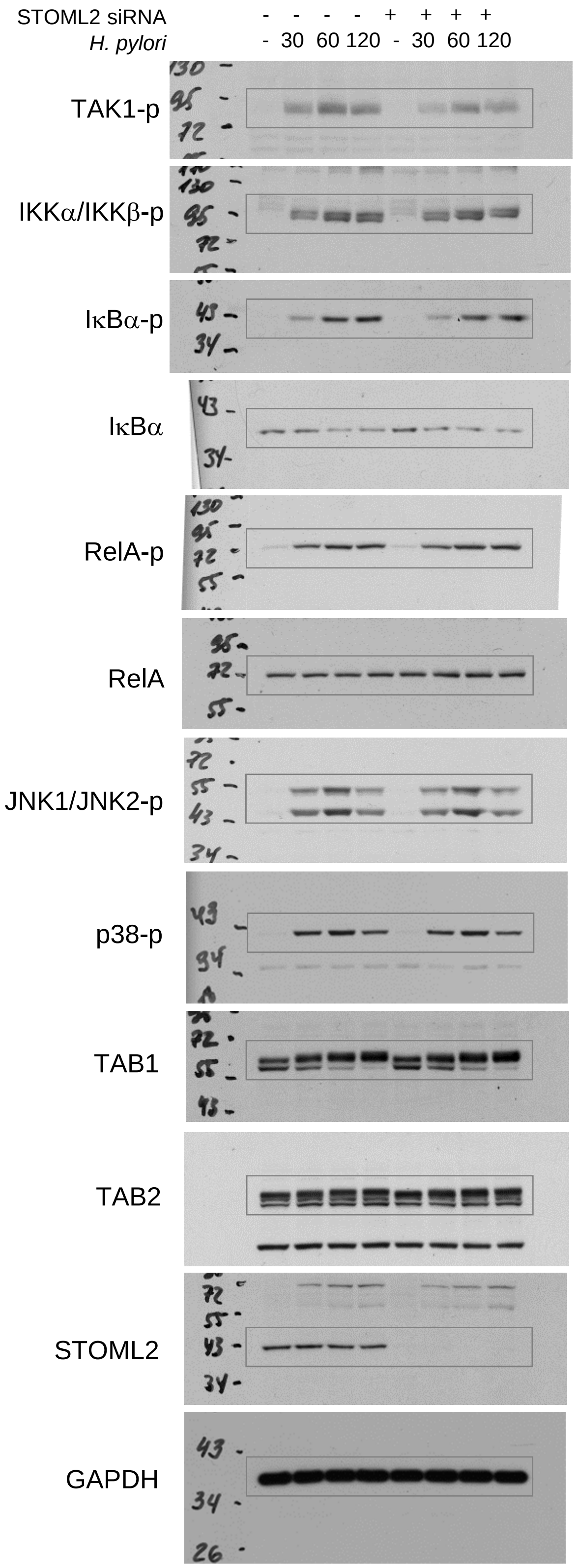
STOML2 siRNA - - - - + + + +
H. pylori - 30 60 120 - 30 60 120
TAK1-p
IKKα/IKKβ-p
IκBα-p
IκBα
RelA-p
RelA
JNK1/JNK2-p
p38-p
TAB1
TAB2
STOML2
GAPDH

C

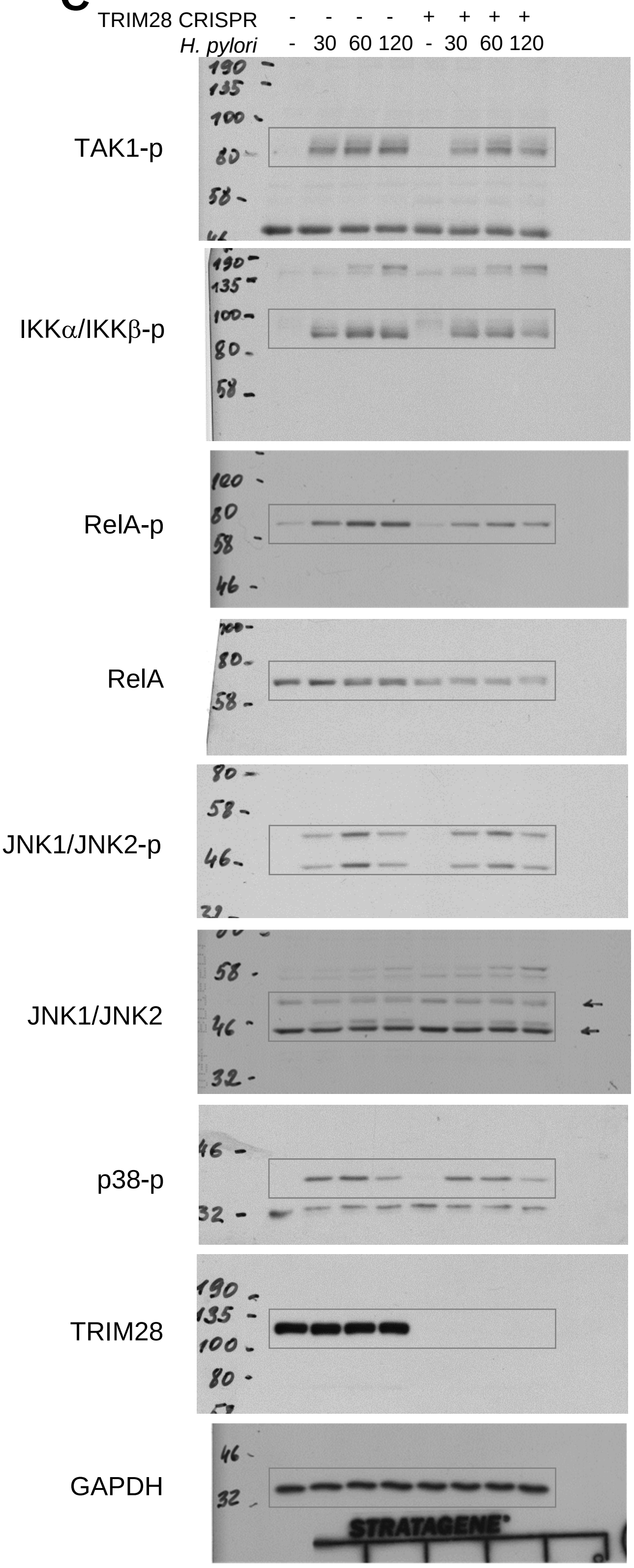
TRIM28 CRISPR - - - - + + + +
H. pylori - 30 60 120 - 30 60 120
TAK1-p
IKKα/IKKβ-p
RelA-p
RelA
JNK1/JNK2-p
JNK1/JNK2
p38-p
TRIM28
GAPDH
STRATAGENE
